# Chromosome-level genome assemblies of *Channa argus* and *Channa maculata* and comparative analysis of their temperature adaptability

**DOI:** 10.1093/gigascience/giab070

**Published:** 2021-10-21

**Authors:** Mi Ou, Rong Huang, Cheng Yang, Bin Gui, Qing Luo, Jian Zhao, Yongming Li, Lanjie Liao, Zuoyan Zhu, Yaping Wang, Kunci Chen

**Affiliations:** Key Laboratory of Tropical and Subtropical Fishery Resources Application and Cultivation, Ministry of Agriculture, Pearl River Fisheries Research Institute, Chinese Academy of Fishery Sciences, Guangzhou 510380, China; State Key Laboratory of Freshwater Ecology and Biotechnology, Institute of Hydrobiology, Chinese Academy of Sciences, Wuhan 430072, China; State Key Laboratory of Freshwater Ecology and Biotechnology, Institute of Hydrobiology, Chinese Academy of Sciences, Wuhan 430072, China; State Key Laboratory of Freshwater Ecology and Biotechnology, Institute of Hydrobiology, Chinese Academy of Sciences, Wuhan 430072, China; Key Laboratory of Tropical and Subtropical Fishery Resources Application and Cultivation, Ministry of Agriculture, Pearl River Fisheries Research Institute, Chinese Academy of Fishery Sciences, Guangzhou 510380, China; Key Laboratory of Tropical and Subtropical Fishery Resources Application and Cultivation, Ministry of Agriculture, Pearl River Fisheries Research Institute, Chinese Academy of Fishery Sciences, Guangzhou 510380, China; State Key Laboratory of Freshwater Ecology and Biotechnology, Institute of Hydrobiology, Chinese Academy of Sciences, Wuhan 430072, China; State Key Laboratory of Freshwater Ecology and Biotechnology, Institute of Hydrobiology, Chinese Academy of Sciences, Wuhan 430072, China; State Key Laboratory of Freshwater Ecology and Biotechnology, Institute of Hydrobiology, Chinese Academy of Sciences, Wuhan 430072, China; State Key Laboratory of Freshwater Ecology and Biotechnology, Institute of Hydrobiology, Chinese Academy of Sciences, Wuhan 430072, China; Innovative Academy of Seed Design, Chinese Academy of Sciences, Beijing 100101, China; Key Laboratory of Tropical and Subtropical Fishery Resources Application and Cultivation, Ministry of Agriculture, Pearl River Fisheries Research Institute, Chinese Academy of Fishery Sciences, Guangzhou 510380, China

**Keywords:** Channa argus, Channa maculata, genome, transcriptome, low-temperature adaptation

## Abstract

**Background:**

*Channa argus* and *Channa maculata* are the main cultured species of the snakehead fish family, Channidae. The relationship between them is close enough that they can mate; however, their temperature adaptability is quite different.

**Results:**

In this study, we sequenced and assembled the whole genomes of *C. argus* and *C. maculata* and obtained chromosome-level genome assemblies of 630.39 and 618.82 Mb, respectively. Contig N50 was 13.20 and 21.73 Mb, and scaffold N50 was 27.66 and 28.37 Mb, with 28,054 and 24,115 coding genes annotated for *C. argus* and *C. maculata*, respectively. Our analyses showed that *C. argus* and *C. maculata* have 24 and 21 chromosomes, respectively. Three pairs of chromosomes in *C. argus* correspond to 3 chromosomes in *C. maculata*, suggesting that 3 chromosomal fusion events occurred in *C. maculata*. Comparative analysis of their gene families showed that some immune-related genes were unique or expandable to *C. maculata*, such as genes related to herpes simplex infection. Analysis of the transcriptome differences related to temperature adaptation revealed that the brain and liver of *C. argus* rapidly produced more differentially expressed genes than *C. maculata*. Genes in the FoxO signalling pathway were significantly enriched in *C. argus* during the cooling process (*P* < 0.05), and the expression of 3 transcription factor genes in this pathway was significantly different between *C. argus* and *C. maculata* (*P* < 0.01).

**Conclusions:**

*C. maculata* may have higher resistance to certain diseases, whereas *C. argus* has a faster and stronger response to low-temperature stress and thus has better adaptability to a low-temperature environment. This study provides a high-quality genome research platform for follow-up studies of Channidae and provides important clues regarding differences in the low-temperature adaptations of fish.

## Background

*Channa argus* (the northern snakehead, NCBI:txid215402, Fishbase ID: 4799) and *Channa maculata* (the blotched snakehead, NCBI:txid188791, Fishbase ID: 8701) are the main cultured species of the snakehead fish family Channidae [1, 2]. In 2019, the output of the snakehead fish in China reached 460,000 tonnes [3]. Snakehead fish are a serious invasive species in North America owing to their ability to rapidly colonize waterways. These fish have a specialized aerial breathing organ, the suprabranchial chamber, which facilitates aquatic-aerial bimodal breathing. This enables them to migrate short distances over land and makes them a good model for research on bimodal breathing [4, 5].

*C. argus* is widely distributed from China, India, and Southeast Asia to the far east of Russia, North Korea, Japan, and other major water systems and has high cold resistance. In contrast, *C. maculata* is distributed in warm water systems in China, the Philippines, Vietnam, Madagascar, the United States, Japan, and other places and has low cold resistance [2]. The ability of fish to adapt to environmental temperature differs as a result of long-term adaptation and evolution, as well as the specific expression of genetic information. The physiological responses of fish to low-temperature exposure have been extensively studied [6], and there is increasing interest in elucidating the mechanisms of fish adaptation to low-temperature environments and tolerance to low-temperature stress at the molecular level [7]. The decreasing costs of high-throughput sequencing and the application of bioinformatics technology have allowed researchers to use omics methods to analyse the molecular mechanisms and signalling pathways of fish responding to low-temperature stress at the overall biological level and explore the functional genes involved in low-temperature tolerance [8].

The cold tolerance of fish is an important economic characteristic of the breed and is related to its growth cycle and extension range [2]. Although the draft genome of *C. argus* has been previously described [9], to better understand the reasons for the difference in cold tolerance between *C. argus* and *C. maculata*, whole-genome sequencing and assembly of both species were carried out in this study. After obtaining chromosome-level genomic sequences of these 2 species, we analysed the transcriptome differences related to temperature adaptation between *C. argus* and *C. maculata*, providing clues for research on the low-temperature adaptation of fish.

## Data Description

### Source of experimental fish and preparation of DNA

A female *C. argus* and a female *C. maculata*, which were provided by the Pearl River Fisheries Research Institute, Chinese Academy of Fishery Sciences, were dissected to obtain muscle tissue and immediately frozen in liquid nitrogen for storage. The cetyltrimethylammonium bromide method was used to extract DNA from the muscle tissue, and 1% agarose gel electrophoresis and Qubit 3.0 (Thermo Fisher Scientific, Inc., Waltham, MA, USA) were used to detect the quality and concentration of the extracted DNA.

Before the dissection of the experimental fish, the fish were anaesthetized with ethyl 3-aminobenzoate methanesulfonate. The experimental protocol of this study was approved by the Animal Ethics Committee of the Institute of Hydrobiology, Chinese Academy of Sciences (reference No.: Y81F101).

### Illumina sequencing and genome survey

Two 350-bp libraries were constructed using the *C. argus* and *C. maculata* muscle tissue DNA, and paired-end 150 bp (PE 150) sequencing was performed on the Illumina NovaSeq 6000 platform (Illumina NovaSeq 6000 Sequencing System, RRID:SCR_016387). The experiments were performed according to the standard protocol provided by Illumina. After the raw data were obtained, 62.90 and 63.90 Gb clean data of *C. argus* and *C. maculata* were obtained by routine filtering. Two *k*-mer distribution maps with *k* = 19 were constructed on the basis of clean data using jellyfish v2.1.4 (Jellyfish, RRID:SCR_005491) (Supplementary File 1: Figure). Based on the distribution of *k*-mers in *C. argus* and *C. maculata*, it was estimated that the content of repeated sequences was 18.73% and 18.23%, and the heterozygosity was 0.12% and 0.06% using genomescope v1.00 (GenomeScope, RRID:SCR_017014) [10], respectively. A total of 49,571,777,400 and 48,531,014,793 *k*-mers of *C. argus* and *C. maculata* were used for genome length estimation, and the calculated genome lengths were 658.63 and 652.03 Mb (the formula is *k*-mer number/average *k*-mer depth), respectively. In addition, according to the sequencing data analysis, the GC contents of *C. argus* and *C. maculata* genomes were 40.36% and 40.37%, respectively. These 2 genomes could be assembled directly because of their compact size, low heterozygosity, and complexity.

### Nanopore sequencing and initial assembly

Two Oxford Nanopore (Oxford Nanopore Technologies, RRID:SCR_003756) long-read libraries were constructed using *C. argus* and *C. maculata* muscle tissue DNA and sequenced on the Nanopore platform. The process was performed using the Ligation Sequencing Kit (catalog number SQK-LSK109; Oxford Nanopore Technologies, Oxford, UK). After filtering out low-quality reads and removing the adapters, 118.24 and 101.34 Gb of clean data were obtained for *C. argus* and *C. maculata*, respectively. The total sequencing depth was 187.57× and 163.76×, the N50 reads were 38.83 and 40.48 kb, and the average read length was 26.59 and 28.11 kb, respectively. Using Canu v1.9 (Canu, RRID:SCR_015880) [11], the clean data were corrected and then assembled using WTDBG v1.2.8 (WTDBG, RRID:SCR_017225) [12], then corrected again with the Nanopore and Illumina sequencing data using Racon (Racon, RRID:SCR_017642) [13] and Pilon v1.23 (Pilon, RRID:SCR_014731) [14], respectively. Finally, the initial assembled genome sequence of *C. argus* and *C. maculata* had a total length of 630.38 and 618.82 Mb, and contig N50 of 21.50 and 23.25 Mb, respectively. Using BWA (BWA, RRID:SCR_010910) [15] to align the Illumina sequencing data with the initial assembled genome, the matching rates were 98.17% and 98.34% (Supplementary File 2: Table). BUSCO v4.0.6 (BUSCO, RRID:SCR_015008) [16] was used to evaluate the integrity of 3,354 conserved core genes in the initial assembled genome, accounting for 93.65% and 97.14%, respectively (Supplementary File 3: Table), indicating that the initial assemblies were useful.

### Super assembly based on Hi-C technology

After fixing and cross-linking the *C. argus* and *C. maculata* muscle tissues with formaldehyde, 2 Hi-C libraries of 300–700 bp were constructed according to the methodology described by Rao et al. [17]. After the libraries were qualified, high-throughput sequencing was performed using an Illumina NovaSeq 6000 with PE150. Raw data were filtered to remove low-quality reads and adapters, and 102.43 and 103.13 Gb clean data for *C. argus* and *C. maculata*, respectively, were obtained. After aligning the clean data with the initial genome assembly, using HiC-Pro v2.11.1 (HiC-Pro, RRID:SCR_017643) [18] to filter the alignment results, 146,400,814 and 151,732,929 valid interaction pairs were obtained. Based on the valid interaction pairs, the initial genome assemblies were further assembled using LACHESIS (LACHESIS, RRID:SCR_017644) [19], including grouping, sorting, and orientation of the initial assembled sequences. Finally, the genome sequences with total lengths of 619.41 and 616.63 Mb were attached to the 24 and 21 chromosomes [20], accounting for 98.26% and 99.65% (619,407,135/630,381,055 and 616,629,265/618,815,250), and the numbers of corresponding sequences were 293 and 227, respectively (Table 1).

**Table 1:** Summary: statistics of the reference genome assemblies of *C. argus* and *C. maculata*

Species	Assembly	Contig No.	Contig length (bp)	Scaffold No.	Scaffold length (bp)
*C. argus*	N50	15	13,290,021	11	27,662,632
	N90	60	1,903,525	22	13,584,876
	Max		28,029,688		50,138,606
	Total	607	630,381,055	521	630,389,655
	Anchored to chromosomes			293	619,407,135 (98.26%)
*C. maculata*	N50	13	21,727,292	9	28,367,461
	N90	44	2,420,044	19	21,794,094
	Max		26,519,478		49,937,344
	Total	338	618,815,250	254	618,823,650
	Anchored to chromosomes			227	616,629,265 (99.65%)

**Figure 1:**
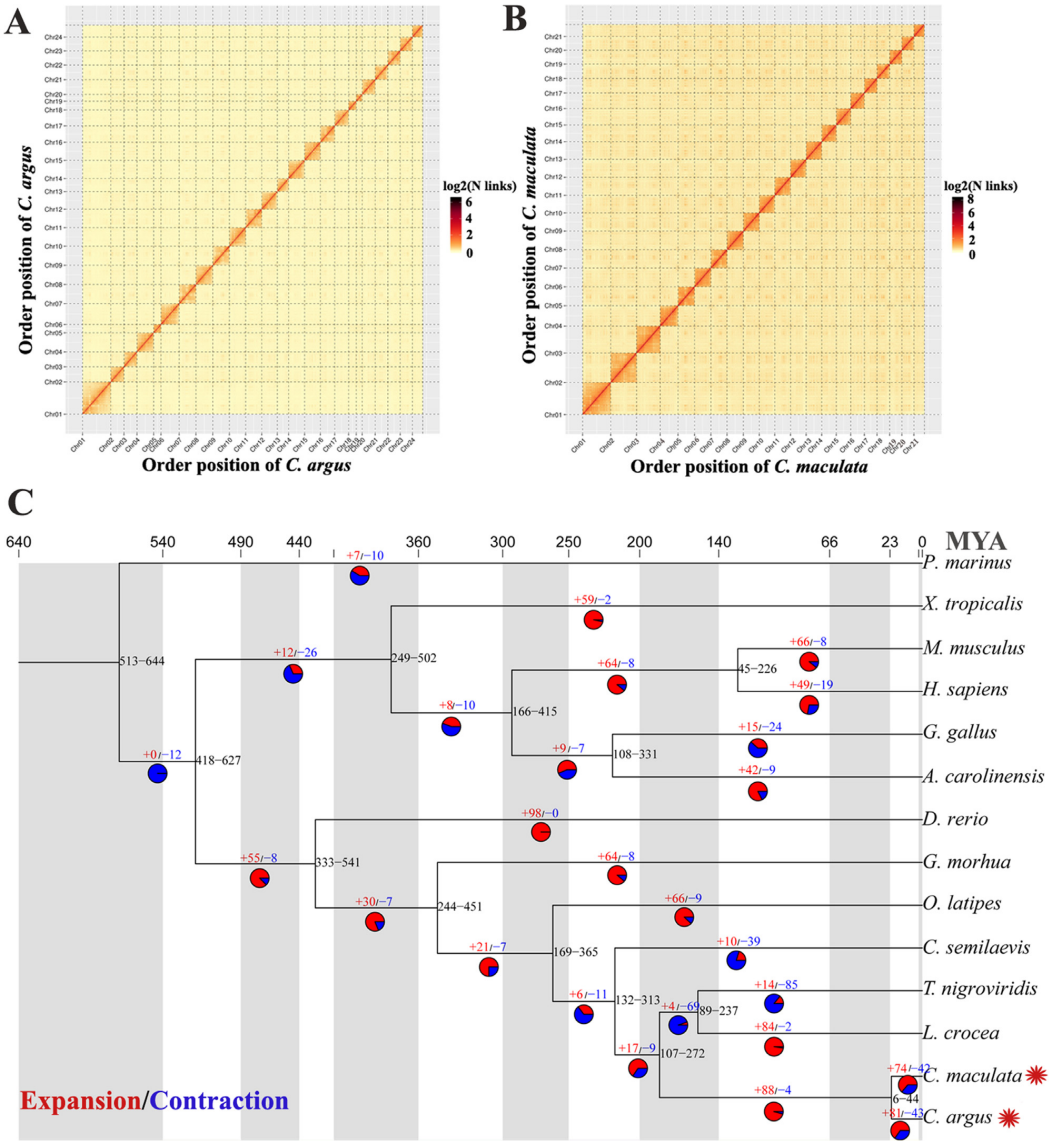
Genome assembly and evolutionary analysis of *C. argus* and *C. maculata*. Genome-wide Hi-C heat maps of *C. argus* (A) and *C. maculata* (B). Chr 1–24 and Chr 1–21 refer to chromosome 1–24 and chromosome 1–21. (C) Evolutionary tree including *C. argus* and *C. maculata*. The black number at each branch represents the divergence time supported by 95% of the highest posterior density (HPD). The top of the tree is absolute age, separated by the shadow of each geological period. The number on the branch shows the number of expanded (red) and contracted (blue) gene families for each clade. The 2 red asterisks indicate *C. argus* and *C. maculata*.

Chromosome-level genomes were cut into 100-kb bins of equal length, and the number of Hi-C read pairs covering any 2 bins was used as the signal of the interaction between the 2 bins. Two heat maps were drawn to evaluate assembly quality (Fig. 1A and B). The image signal distinguished the 24 and 21 chromosome groups, and the intensity of the interaction at the diagonal position on each chromosome was higher than that at the off-diagonal position, indicating that the assembly effect of the chromosomes was strong.

### Annotation of repetitive sequences, coding genes, and non-coding RNA

Using LTR_FINDER (LTR_Finder, RRID:SCR_015247) [21] and RepeatScout v1.0.5 (RepeatScout, RRID:SCR_014653) [22], 2 repetitive sequence databases of the genomes were constructed on the basis of the principles of structure prediction and *de novo* prediction. PASTEClassifier (PASTEClassifier, RRID:SCR_017645) [23] was used to classify the databases, which were then merged with the Repbase (Repbase, RRID:SCR_021169) database [24] as the final repetitive sequence databases. RepeatMasker v4.0.9 (RepeatMasker, RRID:SCR_012954) [25] was then used to predict the repetitive sequences of the genomes based on the constructed repetitive sequence database, with repetitive sequences of 117.49 and 118.99 Mb obtained from *C. argus* and *C. maculata*, respectively (Supplementary File 4: Table).

Genscan (GENSCAN, RRID:SCR_013362) [26], Augustus v2.4 (Augustus, RRID:SCR_008417) [27], GlimmerHMM v3.0.4 (GlimmerHMM, RRID:SCR_002654) [28], GeneID v1.4 (Entrez Gene, RRID:SCR_002473) [29], and SNAP v2006-07-28 (SNAP—SNP Annotation and Proxy Search, RRID:SCR_002127) [30] were used for *de novo* prediction of coding genes. GeMoMa v1.3.1 (GeMoMa, RRID:SCR_017646) [31, 32] was used for predictions based on homologous species. Hisat v2.0.4 (HISAT2, RRID:SCR_015530) [33] and Stringtie v1.2.3 (StringTie, RRID:SCR_016323) [34] were used to assemble transcripts with reference sequences, and TransDecoder v2.0 (TransDecoder, RRID:SCR_017647) [35] and GeneMarkS-T v5.1 (GeneMarkS-T, RRID:SCR_017648) [36] were used to perform gene prediction. PASA v2.0.2 (PASA, RRID:SCR_014656) [37] was used to predict unigene sequences based on transcriptome data without reference sequences. EVM v1.1.1 (EVidenceModeler, RRID:SCR_014659) [38] was used to integrate the prediction results obtained from the above methods and was modified with PASA v2.0.2. Finally, 28,054 and 24,115 coding genes in *C. argus* and *C. maculata* were predicted (Supplementary File 5: Table). The number of genes supported by the 3 prediction methods *ab initio*, homology, and RNAseq was 20,544 and 19,990, accounting for 73.23% (20,544/28,054) and 82.89% (19,990/24,115) for *C. argus* and *C. maculata*, respectively.

Different strategies have been used to predict different non-coding RNAs based on the structural characteristics of different non-coding RNAs. Using the Rfam (Rfam, RRID:SCR_007891) database [39], BLASTN (BLASTN, RRID:SCR_001598) was used to perform genome-wide alignment to identify microRNAs and ribosomal RNAs. Transfer RNAs (tRNAs) were identified using tRNAscan-SE v2.0 (tRNAscan-SE, RRID:SCR_010835) [40]. Finally, a total of 554 and 247 microRNAs, 1,136 and 633 ribosomal RNAs, and 4,172 and 1,784 tRNAs were predicted in *C. argus* and *C. maculata*, respectively (Supplementary File 6: Table).

### Whole-genome evolutionary analysis

The genome data of 14 vertebrate species spanning amphibians to mammals with different evolutionary relationships (including *C. argus* and *C. maculata*) were compared. Using Orthofinder v2.3.7 (OrthoFinder, RRID:SCR_017118) [41], the protein sequences of these 14 species were classified into families, and the PANTHER v15 (PANTHER, RRID:SCR_004869) database [42] was used to annotate the obtained gene families. A total of 30,269 families were obtained, of which 1,023 were single-copy gene families. A total of 858 families were unique to *C. argus*, and 46 families were unique to *C. maculata* (Supplementary File 7: Table). Using the 1,023 single-copy gene families and IQ-TREE v1.6.11 (IQ-TREE, RRID:SCR_017254) [43], an evolutionary tree was constructed using the maximum likelihood (ML) method with the number of bootstraps set to 1,000 and the outgroup set to *Petromyzon marinus*. PAML v4.9i (PAML, RRID:SCR_014932) [44] was used to calculate the divergence time, and MCMCtreeR v1.1 (PAML, RRID:SCR_014932) [45] was used for the evolutionary tree display (Fig. 1C). The genetic relationship between *C. argus* and *C. maculata*, belonging to Perciformes, was the closest, and the differentiation time was 6–44 million years ago (MYA).

**Figure 2:**
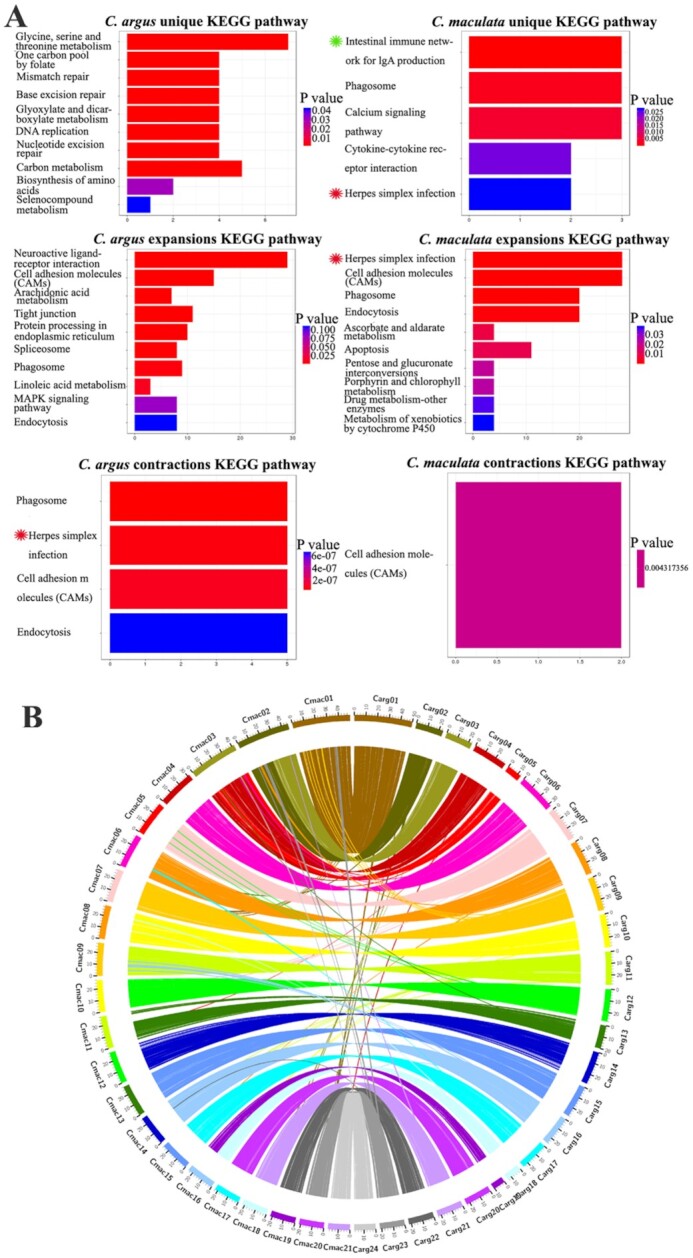
Comparative analysis of the *C. argus* and the *C. maculata* genomes. (A) KEGG enrichment analysis of the unique, expansion, and contraction gene families. The ordinate is KEGG terms, the abscissa is the number of genes in the pathway, and the colour represents the corresponding *P*-value. *Left*, enrichment result for *C. argus; right*, enrichment result for *C. maculata*, same asterisks indicate same terms. (B) There was a high collinearity between the 2 species. Chr 2 and 3 of *C. argus* correspond to Chr 2 of *C. maculata*, Chr 4 and 5 of *C. argus* correspond to Chr 3 of *C. maculata*, Chr 18 and 19 of *C. argus* correspond to Chr 16 of *C. maculata*.

On the basis of the phylogenetic tree showing divergence time and the results of gene family clustering, the number of ancestral gene family members of each branch was estimated using CAFE v4.2 (CAFE, RRID:SCR_005983) [46], so as to predict the expansion and contraction of the gene family relative to its ancestors (*P* < 0.05) (Supplementary File 8: Table). The results showed that there were 81 expanded gene families, including 606 genes, and 43 contracted gene families, including 95 genes in *C. argus*, while *C. maculata* contained 74 expanded gene families, including 721 genes, and 42 contracted gene families, including 8 genes. GO and KEGG enrichment analyses were performed using clusterProfile v3.5.1 (clusterProfiler, RRID:SCR_016884) (Fig. 2A, Supplementary File 9: Figure). The results showed that there were specific immune pathway–related genes in *C. maculata* compared to *C. argus*, such as the genes involved in the intestinal immune network for lgA production and the genes related to the herpes simplex infection pathway. In addition, the members of the herpes simplex infection gene family in *C. maculata* showed significant expansion (*P* < 0.05).

**Figure 3:**
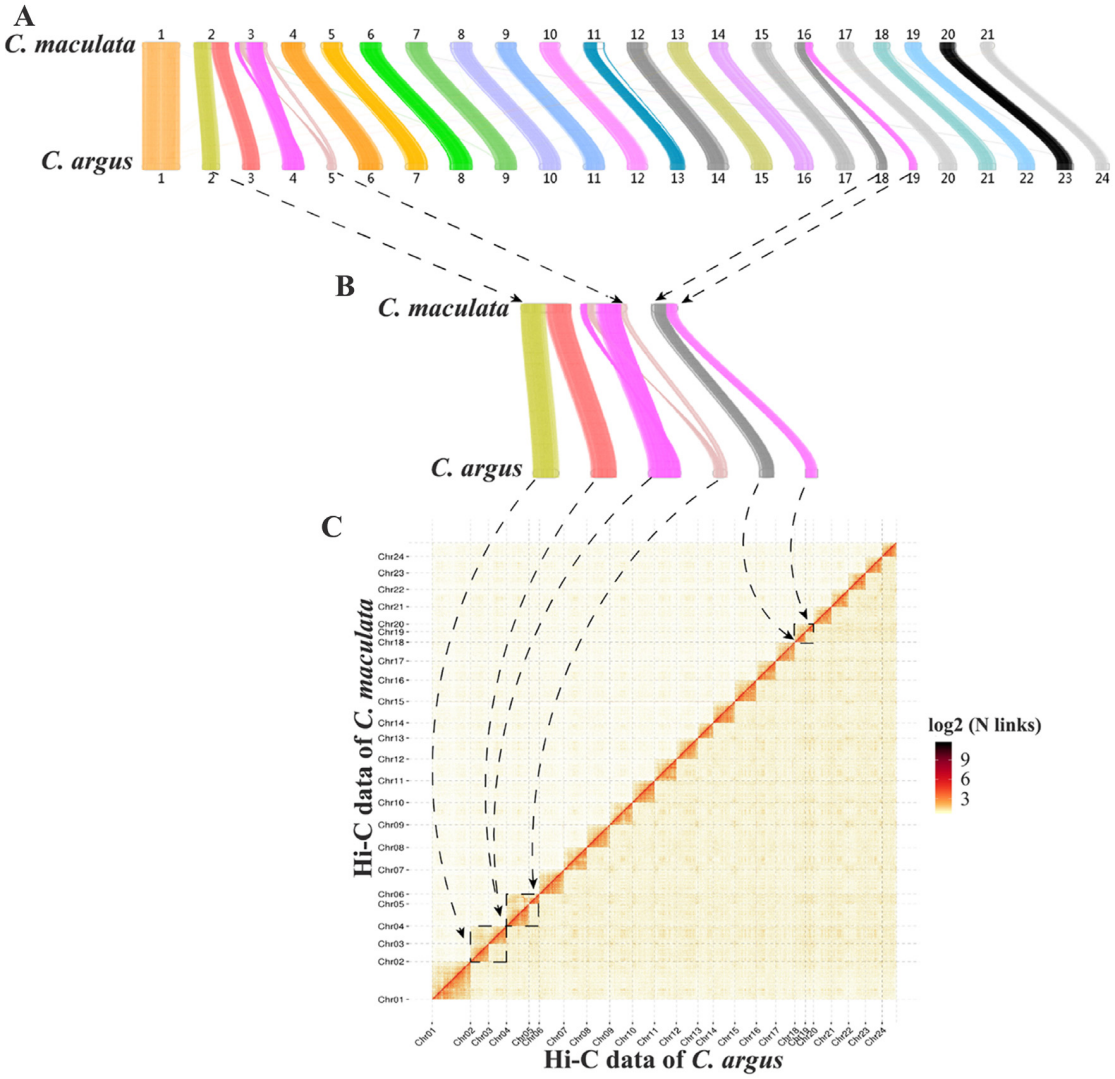
Verification of chromosome structure differences between *C. argus* and *C. maculata* genomes. (A) Complete collinearity map. (B) Partial collinearity map showing only the chromosomes with structural differences. (C) *C. argus* chromosomes were set as the reference sequence to which the Hi-C data of *C. argus* and *C. maculata* were mapped.

Using BLASTP (BLASTP, RRID:SCR_001010) to compare the gene protein sequences of these 2 species, the genes in all collinearity blocks were obtained, and the collinearity map of the coding genes of *C. argus* and *C. maculata* was drawn using MCScanX [47] (Fig. 2B). Chr 2 and 3 of *C. argus* correspond to Chr 2 of *C. maculata*, Chr 4 and 5 of *C. argus* correspond to Chr 3 of *C. maculata*, and Chr 18 and 19 of *C. argus* correspond to Chr 16 of *C. maculata*. Using the 24 chromosomes of *C. argus* as a reference, the Hi-C data of *C. argus* and *C. maculata* were mapped to it, and the mapping results confirmed the structural differences (Fig. 3).

### Low-temperature stress and transcriptome sequencing

**Figure 4:**
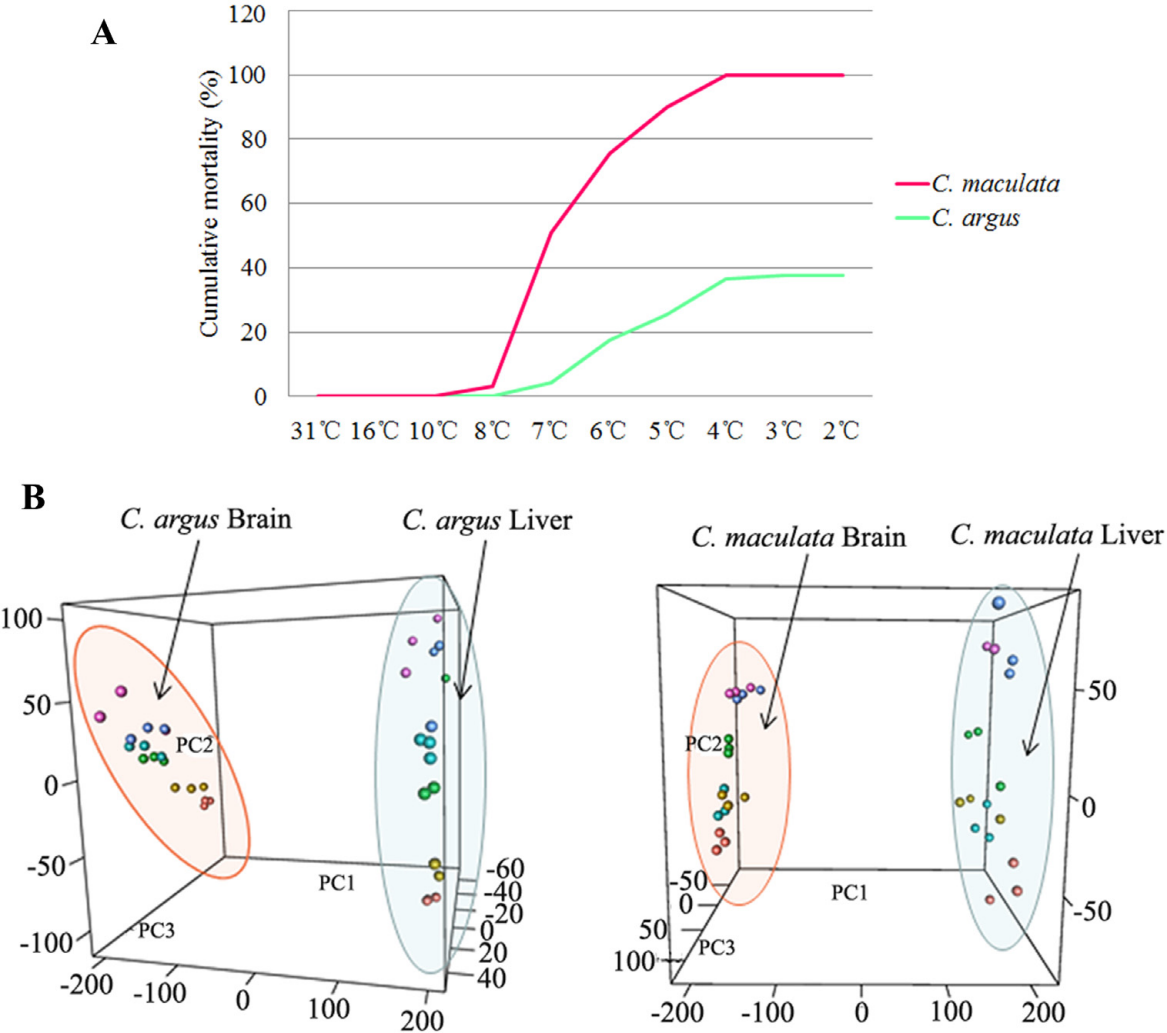
Low-temperature experiment and transcriptome sequencing of *C. argus* and *C. maculata*. (A) Cumulative mortality of *C. argus* and *C. maculata* during cooling. Abscissa represents temperature and ordinate represents cumulative mortality. (B) Principal component analysis (PCA) of expression genes in brain and liver at different temperatures. Coordinates are the first 3 principal components PC1, PC2, and PC3 of PCA, and the scale value represents the contribution of the sample to the principal component.

A total of 180 *C. argus* and *C. maculata* specimens (aged 2 months), weighing 86 ± 17 and 56 ± 9 g, were placed in 2 barrels of 700-L capacity, 90 in each barrel. One group was monitored for observation and statistical mortality, while the other was used to collect materials. The fish were kept at 31°C for 2 weeks. Subsequently, the circulating water-cooling device was connected, and the temperature gradually decreased (Supplementary File 10: Table). During this process, the status and mortality of *C. argus* and *C. maculata* were recorded daily (Supplementary File 10: Table), and a cumulative mortality map was drawn (Fig. 4). *C. argus* began to die at 7°C, and 34 died at 7–2°C, with a mortality rate of 38% (34/90). No deaths occurred in the following 3 days. *C. maculata* began to die at 8°C, peaking at 7°C, and all specimens died at 8–4°C, with a mortality rate of 100% (Fig. 4A). Three *C. argus* and *C. maculata* were randomly selected before cooling (31°C), and brain and liver tissues were collected from each fish. During the cooling period, samples were collected after the temperature was maintained at 16°C for 24 h, and again at 10°C, 8°C, 6°C, and 4°C. Sampling took place before 8:00 (before cooling) on the day, with brain and liver tissue from 3 specimens for each species collected at each time point.

After completion of the low-temperature stress, 72 tissue samples (6 time points, 3 *C. argus* and 3 *C. maculata*, 2 tissues per fish) were collected for transcriptome sequencing (PE 150). The sequencing platform was an Illumina NovaSeq 6000, and each sample produced ≥6 Gb of clean data.

### Statistical analysis of transcriptional sequencing data and expressed genes

The data obtained from each tissue are shown in Supplementary File 11: Table. Using hisat2 (HISAT2, RRID:SCR_015530) [48], clean reads from each tissue were aligned with the genomes of *C. argus* and *C. maculata*. After the initial treatment of the gene count matrix by rlogTransformation of DEseq2 (DESeq2, RRID:SCR_015687) [49], the gene expression density map of the normalized genes showed that the gene expression in brain and liver tissues of *C. argus* and *C. maculata* was negative binomial (Supplementary File 12: Figure A and Supplementary File 13: Figure A).

The transcripts per million (TPM) of each gene were calculated, and the genes of TPM > 1 in all samples were counted. Based on this, we drew the box line and cluster diagrams, and a PCA map of tissue expression to analyse the overall expression of the genes and the correlations between tissues (Supplementary File 12: Figure B, C, Supplementary File 13: Figure B, C, and Fig. 4B). The box line diagram showed that the number of genes detected in the brain tissue of *C. argus* and *C. maculata* was obviously higher than that in the liver (Supplementary File 12: Figure B and Supplementary File 13: Figure B). PCA and cluster analysis showed that the difference between the brain and liver in *C. argus* and *C. maculata* was the most significant variable (∼75%) in gene expression, and the change in temperature was the second largest variable in PCA, accounting for 4%–6% of the total variable.

### Differential expression analysis of genes

**Figure 5:**
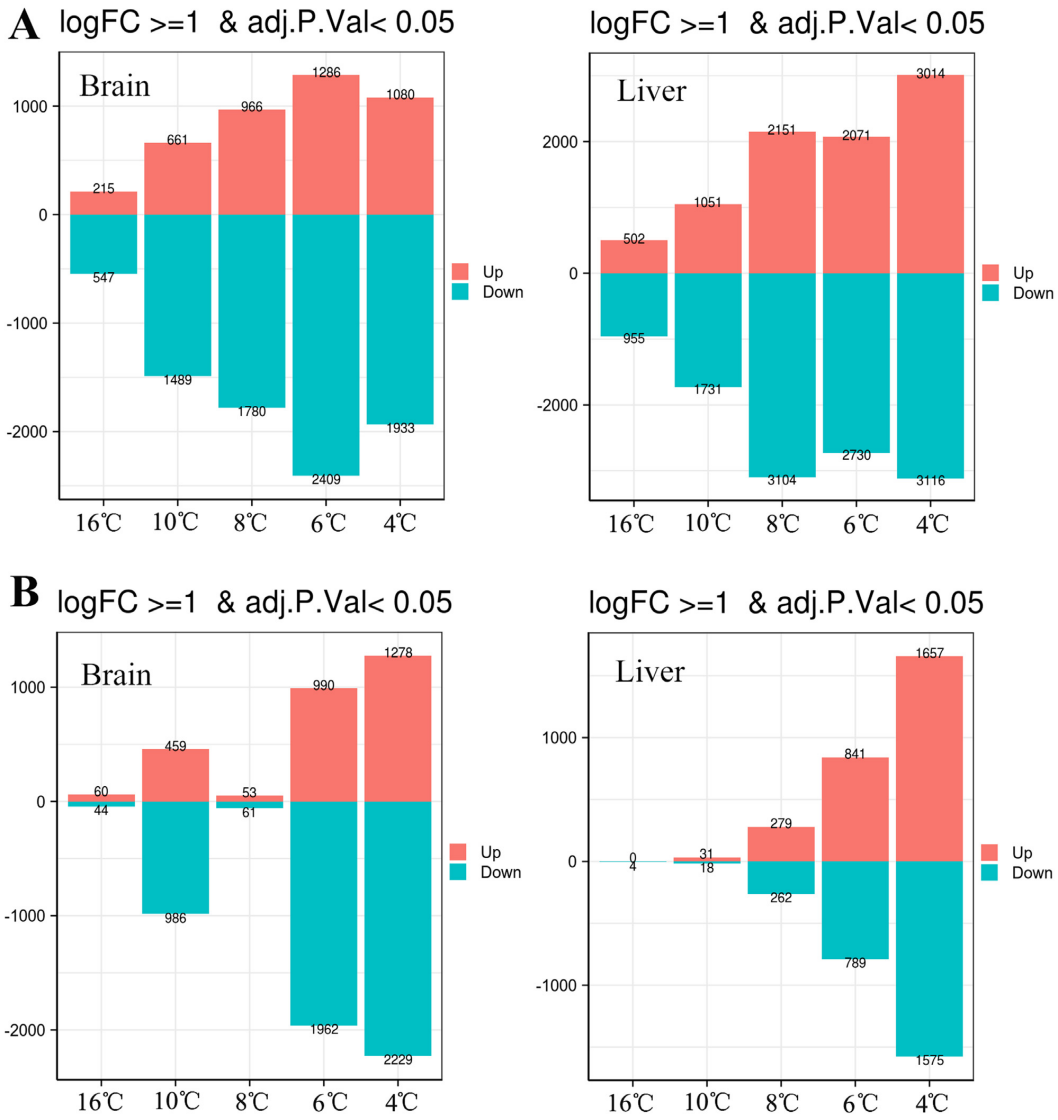
Number of DEGs in brain and liver of *C. argus* (A) and *C. maculata* (B) during cooling. The abscissa represents temperature and the ordinate represents the number of genes. Red indicates up-regulated genes and blue indicates down-regulated genes. FC: fold change.

The number of differentially expressed genes (DEGs) with log fold-change ≥1 at each time point was counted, with the gene expression level at the control temperature (31°C) set as the baseline control (Fig. 5). As the temperature decreased, the number of DEGs in the brain and liver of *C. argus* increased rapidly. At 16°C, genes in the brain and liver were obviously upregulated and downregulated. At 4°C, the number of DEGs in the brain began to decrease, while the opposite response was observed in the liver. In *C. maculata*, the number of DEGs in the brain increased considerably at 10°C. At 8°C, the number of DEGs in the brain of *C. maculata* suddenly decreased to the same level as that observed at 16°C, which may be related to the phenotypic characteristics of death and massive shock that occurred at 8°C (Supplementary File 10: Table).

**Figure 6:**
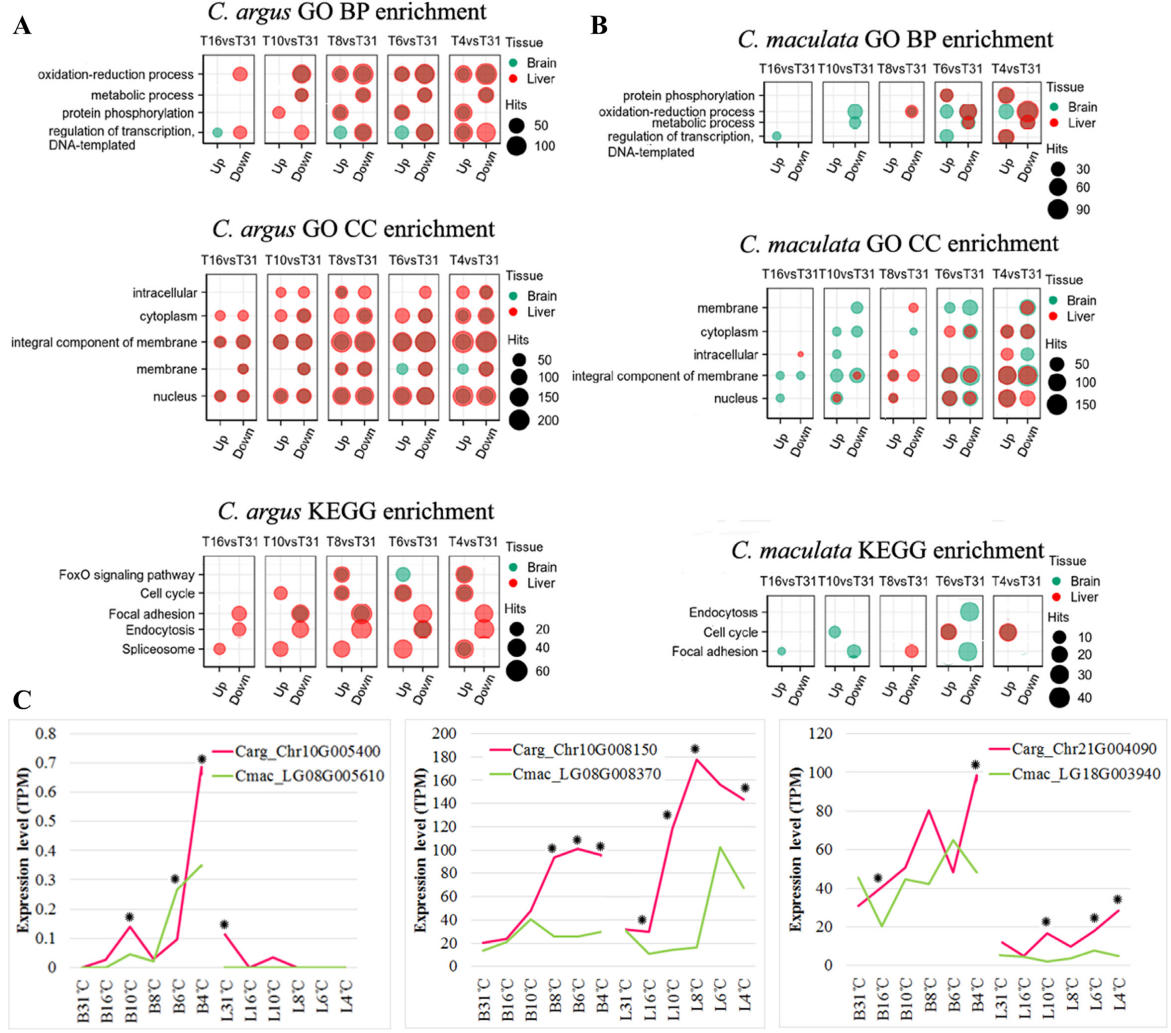
GO and KEGG enrichment analysis of DEGs. The genes with noticeable differences between *C. argus* and *C. maculata* were selected for display. (A) Enrichment results for *C. argus* (green for brain, red for liver). The area of the circle indicates the number of genes. (B) Enrichment results for *C. maculata*. (C) The expression of 3 transcription factor genes in *C. argus* and *C. maculata*. The abscissa represents the tissue samples at different temperatures, and the ordinate represents the expression quantity. The asterisk indicates a significant difference (*P* < 0.01).

DEG enrichment at each time point was assessed by GO and KEGG analyses, and the top 5 significantly enriched items (*P* < 0.05) were selected for illustration. It was found that the functions of DEGs were mainly involved in oxidation-reduction processes, metabolic processes, protein phosphorylation, and the pathways mainly involved the FoxO signalling pathway, cell cycle, focal adhesions, and so forth (Fig. 6A and B). We noticed that the FoxO signalling pathway only appeared in the top 5 items for *C. argus*. The FoxO signalling pathway is a transcription factor–related signalling pathway (Fig. 6A). We selected all 88 genes enriched in the FoxO signalling pathway in *C. argus*, and iTAK [50] predicted that 10 of these were transcription factors. Based on the collinear relationship of genes between *C. argus* and *C. maculata*, we identified 10 corresponding genes in *C. maculatus* (Supplementary File 14: Table). Transcriptome data were used to analyse the expression changes of 10 transcription factor genes during the cooling process, and it was found that 3 showed significant differences between *C. argus* and *C. maculata* (*P* < 0.01) (Fig. 6C). It is speculated that these genes may be involved in the regulation of cold tolerance traits in *C. argus*.

## Conclusion

In this study, we sequenced the whole genome of 2 Channidae fish, *C. argus* and *C. maculata*, and assembled genome sequences at the chromosome level, which can provide a high-quality genome research platform for follow-up research. The contig N50 was 13.20 and 21.73 Mb, and the scaffold N50 was 27.66 and 28.37 Mb for *C. argus* and *C. maculata*, respectively. Compared with the previously published draft genome of *C. argus*, which had a contig N50 of 81.4 kb and a scaffold N50 of 4.5 Mb [9], the quality of the genomes obtained in this study represents a substantial improvement.

Genome comparison analysis revealed that *C. maculata* contains genes involved in the intestinal immune network for lgA production and the herpes simplex infection pathway that are not present in *C. argus*. In addition, members of the herpes simplex infection gene family also have a significant expansion in *C. maculata*. Compared with *C. argus, C. maculata* may have higher resistance to disease, especially herpes simplex infection.

There are 3 pairs of chromosomes in *C. argus* that correspond to 3 chromosomes in *C. maculata*. The median number of chromosomes in fish with known chromosome number is 24 [51–53]. Therefore, we speculate that these chromosomes in *C. maculata* fused, while those in *C. argus* did not.

This study carried out transcriptome analysis to analyse why the cold tolerance of *C. argus* is better than that of *C. maculata*. It is found that both *C. argus* and *C. maculata* had obvious up-regulation and down-regulation responses in oxidation-reduction processes, metabolic processes, protein phosphorylation, and other pathways, representing the core molecular response to low-temperature exposure. However, a key difference was that the brain and liver of *C. argus* quickly produced more DEGs, indicating that the response of *C. argus* to low temperature was faster and stronger than that of *C. maculata*. In many organisms, transcriptional regulation is a direct response to cold environments. Cold-adapted fish rely on special strategies to acclimate to cold conditions, such as protein biosynthesis, energy metabolism, immune system, lipid metabolism, and signalling pathways, and these strategies have been proven to be species-specific [54]. The FoxO transcription factor–related signalling pathway was significantly enriched in *C. argus* (*P* < 0.05) (Fig. 6A). The FoxO family of transcription factors regulates the expression of genes involved in cellular physiological events including apoptosis, cell cycle control, glucose metabolism, oxidative stress resistance, and longevity [55]. Three genes in this pathway showed significant differential expression between *C. argus* and *C. maculata* (Fig. 6C), and their function in low-temperature adaptation requires further accurate verification and analysis.

## Data Availability

Genome, annotation files, and raw sequences for genome assembly including Illumina, Nanopore, and Hi-C reads of *C. argus* were deposited in the NCBI and can be accessed with accession No. PRJNA731586, and the corresponding data of *C. maculata* can be accessed with accession No. PRJNA730430. The transcriptome data related to temperature adaptation of *C. argus* and *C. maculata* can be accessed with accession No. PRJNA732763. Supporting data and materials are available in the* GigaDB* database [56], with individual datasets for *C. argus* [57] and *C. maculata* [58].

## Additional Files

**Supplementary File 1**: **Figure**. *k*-mer distribution of reads of *C. argus* (A) and *C. maculata* (B). *k*-mers (*k* = 19) were extracted from the paired-end library with an insert size of 350 bp. The peak 19-mer depths were 76 (A) and 75 (B), respectively.

**Supplementary File 2**: **Table**. Mapping rates of the Illumina sequencing data.

**Supplementary File 3**:**Table**. Integrity of 3.354 conserved core genes.

**Supplementary File 4**: **Table**. Annotation of repetitive sequences.

**Supplementary File 5**: **Table**. Annotation of coding genes.

**Supplementary File 6**: **Table**. Annotation of non-coding RNA.

**Supplementary File 7**: **Table**. Single-copy genes and specific genes in *C. argus* and *C. maculata*.

**Supplementary File 8**: **Table**. Gene family statistics for expansion and contraction.

**Supplementary File 9**: **Figure**. GO enrichment analysis of genes in expansion/contraction families. (A, B) Results for *C. argus*. (C, D) Results for *C. maculata*. The abscissa represents the GO terms, and the ordinate represents the number and percentage of genes. Ten GO terms with the most significant enrichment were selected and displayed.

**Supplementary File 10**: **Table**. Status and mortality of *C. argus* and *C. maculata* during cooling.

**Supplementary File 11**: **Table**. Statistical data of transcriptome sequencing.

**Supplementary File 12**: **Figure**. Preliminary analysis of *C. argus* sequencing data. (A) Gene expression in the brain and liver showed a negative binomial distribution. The abscissa represents the log_2_ value of the amount of gene expression, and the ordinate represents the percentage. (B) The box line diagram shows that the number of genes detected in the brain was higher than that in the liver. The abscissa represents the tissue and the ordinate represents the number of genes. (C) Cluster diagram of brain and liver at different temperatures. Different font colours indicate different temperatures.

**Supplementary File 13**: **Figure**. Preliminary analysis of *C. maculata* sequencing data. (A) Gene expression in the brain and liver showed a negative binomial distribution. The abscissa represents the log_2_ value of the amount of gene expression, and the ordinate represents the percentage. (B) The box line diagram shows that the number of genes detected in the brain was higher than that in the liver. The abscissa represents the tissue and the ordinate represents the number of genes. (C) Cluster diagram of brain and liver at different temperatures. Different font colours indicate different temperatures.

**Supplementary File 14**: **Table**. Ten transcription factor genes in the FoxO signalling pathway in *C. argus* and *C. maculata*.

## Abbreviations

BLAST: Basic Local Alignment Search Tool; BWA: Burrows-Wheeler Aligner; DEGs: differentially expressed genes; Gb: gigabase pairs; GC: guanine cytosine; GO: gene ontogeny; kb: kilobase pairs; KEGG: Kyoto Encyclopedia of Genes and Genomes; Mb: megabase pairs; ML: maximum likelihood; MYA: million years ago; NCBI: National Center for Biotechnology Information; PCA: principal component analysis; PE: paired end; SRA: Sequence Read Archive; TPM: transcripts per million.

## Competing Interests

The authors declare that they have no competing interests.

## Funding

This work was supported by the National Key Research & Development Program of China(2018YFD0901201) and the State of Key Laboratory of Freshwater Ecology and Biotechnology (2019FBZ05).

## Authors’ Contributions

K.C. and Y.W. conceived and designed the experiments. M.O., R.H., and B.G. performed the experiments. C.Y., Q.L., J.Z., and L.L. analysed the genome and transcriptome data. R.H., M.O., and Y.L. drafted the manuscript. Y.W. and Z.Z. provided advice on manuscript writing. All authors reviewed and declare that they have no conflict of interest.

## Supplementary Material

giab070_GIGA-D-21-00172_Original_Submission

giab070_GIGA-D-21-00172_Revision_1

giab070_GIGA-D-21-00172_Revision_2

giab070_GIGA-D-21-00172_Revision_3

giab070_Response_to_Reviewer_Comments_Original_Submission

giab070_Response_to_Reviewer_Comments_Revision_1

giab070_Response_to_Reviewer_Comments_Revision_2

giab070_Reviewer_1_Report_Original_Submission Nicolas Rohner -- 6/18/2021 Reviewed

giab070_Reviewer_2_Report_Original_Submission Nansheng (Jack) Chen -- 7/7/2021 Reviewed

giab070_Supplemental_Files
