# Supplementary material for: Chromosome-level genome assemblies of Channa argus and Channa maculata and comparative analysis of their temperature adaptability
Source: Gigascience. 2021 Oct 21;10(10):giab070. doi: 10.1093/gigascience/giab070 (PMC8529964; doi:10.1093/gigascience/giab070)
Supplement: giab070_GIGA-D-21-00172_Revision_1 [file giab070_giga-d-21-00172_revision_1.pdf]

GigaScience

Chromosome-level genome assemblies of C. argus and C. maculata and comparative analysis of their temperature adaptability

--Manuscript Draft--

**Manuscript Number:**	GIGA-D-21-00172R1
**Full Title:**	Chromosome-level genome assemblies of C. argus and C. maculata and comparative analysis of their temperature adaptability
**Article Type:**	Data Note
**Funding Information:**	the National Key Research & Development Program of China (2018YFD0901201) — Mr. Kunci Chen<br>State Key Laboratory of Desert and Oasis Ecology (2019FBZ05) — Mr. Yaping Wang
**Abstract:**	Background: Channa argus and Channa maculata are the main cultured species of the family Channidae. The relationship between them is close enough that they can mate, however their temperature adaptability is quite different. Results: In this study, we sequenced and assembled the whole genomes of C. argus and C. maculata for the first time and obtained chromosome-level genome assemblies of 630.39 and 618.82 Mb, respectively. Contig N50 was 13.20 and 21.73 Mb, scaffold N50 was 27.66 and 28.37 Mb, with 28,054 and 24,115 coding genes annotated for C. argus and C. maculata, respectively. C. argus and C. maculata have 24 and 21chromosomes, respectively. Three pairs of chromosomes in C. argus correspond to three chromosomes in C. maculate, suggesting three chromosomal fusion events in C. maculate. Comparative analysis of their gene families showed that some immune-related genes were unique or expandable to C. maculate, such as genes related to herpes simplex infection. The transcriptome differences related to temperature adaptation revealed that the brain and liver of C. argus rapidly produced more DEGs than C. maculata. The genes in the FoxO signalling pathway were significantly enriched in C. argus during the cooling process (P < 0.05), and the expression of three transcription factor genes in this pathway was significantly different between C. argus and C. maculata (P < 0.01). Conclusions: C. maculata may have higher resistance to certain diseases, while C. argus has a faster and stronger response to low-temperature stress, and thus has better adaptability to a low-temperature environment. This study provides a high-quality genome research platform for follow-up studies of Channidae, and provides important clues for the differences in the low-temperature adaptation of fish.
**Corresponding Author:**	亚平 汪<br>CAS IHB: Institute of Hydrobiology Chinese Academy of Sciences<br>Wuhan, CHINA
**Corresponding Author Secondary Information:**	
**Corresponding Author's Institution:**	CAS IHB: Institute of Hydrobiology Chinese Academy of Sciences
**Corresponding Author's Secondary Institution:**	
**First Author:**	Yaping Wang
**First Author Secondary Information:**	
**Order of Authors:**	Yaping Wang<br>Mi Ou<br>Rong Huang<br>Cheng Yang<br>Bin Gui

	Qing Luo
	Jian Zhao
	Yongming Li
	Lanjie Liao
	Zuoyan Zhu
	Kunci Chen
**Order of Authors Secondary Information:**	
**Response to Reviewers:**	Reviewer reports:<br><br>Reviewer #1: The manuscript: "Chromosome-level genome assemblies of C. argus and C. maculate and comparative analysis of their temperature adaptability" by Ou and Huang et al. provides high quality genomes of two fish species that show differences in their cold tolerance. Using transcriptomic data of tissues collected from fish maintained under cold stress, the authors provide some insights into the pathways that are potentially implicated in this physiological adaptation. I have no major issues with the work and believe it will be of interest to the readers of GigaScience.<br>Answer: Thank you for your professional review!<br><br>Reviewer #2: The manuscript by Ou et al., described the construction of high-quality genomes for two fish species Channa argus and Channa maculata. These species have high economic importance as well as important difference in adaptation to cold. This manuscirpt is valuable because of the high-quality of the genome assembilies, intresting differences in the chromosomal structures (with three independent fusion events), and important phsiological differences of these two species. The publication of this manuscript would facilitate further research on molecular mechanisms underlying the differential adaptation to cold. However, before the paper is accepted for publication, many minor errors, most of which were typos and grammatical errors, need to be addressed.<br><br>1. "Genomic collinearity showed that three pairs of chromosomes in C. argus correspond to three chromosomes in C. maculata." This sentence is unclear. "Based on the above results, it is speculated that the chromosomes of C. maculata fused during evolution". This sentence is not supported. Should be modified as something like this: C. maculata and C. argus have 21 and 24 chromosomes, respectivley. Three pairs of chromosomes in C. argus correspond to three chromosomes in C. maculata, suggesting three chromsomal fusion events in C. maculata, or three chromosomal splitting in C. argus.<br>Answer: Thank you for your suggestion and patient modification! We have revised it according to the suggestion. Please see the blue font section of the abstract.<br><br>2. "A female C. argus and a female C. maculata provided by the Pearl River Fisheries Research Institute, Chinese Academy of Fishery Sciences, were dissected to obtain muscle tissue and immediately frozen in liquid nitrogen for storage." Should be modified as: ""A female C. argus and a female C. maculata, which were provided by the Pearl River Fisheries Research Institute, Chinese Academy of Fishery Sciences, were dissected to obtain muscle tissue and immediately frozen in liquid nitrogen for storage."<br>Answer: We have revised it according to the suggestion. Please see the Data Description 1.<br><br>3. "The experimental procedure was performed according to the standard protocol provided by Illumina." Should be modified as: "The experiment was performed according to the standard protocol provided by Illumina."<br>Answer: We have revised it according to the suggestion. Please see the Data Description 2.<br><br>4. Contig -> contig<br>Answer: Thank you for your reminding. We have revised it according to the suggestion. Please see the Data Description 3.

Powered by Editorial Manager® and ProduXion Manager® from Aries Systems Corporation

	5. "From amphibians to mammals, 14 species (including C. argus and C. maculata) with different evolutionary degrees were collected". Should be modified as "From amphibians to mammals, genome data of 14 species (including C. argus and C. maculata) with different evolutionary relationships were collected."<br>Answer: We have revised it according to the suggestion. Please see the Data Description 6.<br><br>6. alligned -> aligned<br>Answer: We have revised it according to the suggestion. Please see the Data Description 8.<br><br>7. PacBio -> Nanopore<br>Answer: Thank you for your suggestions. We have revised it according to the suggestion. Please see the Data Availability.<br><br>Reviewer #3: This work sounds good. I have some comments as followed.<br>1.It should be give much more introductions on the research progress of temperature adaptability.<br>Answer: Thank you for your careful review and suggestions. We have added relevant contents in the background, please see the second paragraph of the background.<br><br>2.'PCA and cluster analysis showed that the difference between the brain and liver in C. argus and C. maculata was the most significant variable (about 75%) in gene expression, and the change of temperature was the second largest variable in PCA, accounting for 4-6% of the total variable.' The differences in gene expression between tissues are obvious, and this study needs more detailed information to explain the differences in different temperature groups.<br>Answer: We have added detailed information to explain the differences in different temperature groups. Please see the third paragraph of the conclusion.<br><br>3.'At 8 °C, the number of DEGs in the brain suddenly decreased to the same level as that at 16 °C, which may be related to the phenotypic characteristics of death and massive shock at 8 °C.' The study didn't elucidate the interesting change.<br>Answer: Thank you for your reminding. This phenomenon is described in additional file 10. We have referenced "Additional file 10" to this place, please see the Data Description 9.<br><br>4.'It was found that the brain and liver of C. argus quickly produced more DEGs, indicating that the response of C. argus to low temperature was faster and stronger than that of C. maculata.' 'Three genes in this pathway showed significant differential expression between C. argus and C. maculata, and their function in low-temperature adaptation requires further accurate verification and analysis.' It is too weak to elucidate the difference of temperature adaptability between C. argus and C. maculate just by the number of DEGs and three genes which are not directly associated with temperature adaptability.<br>Answer: Thank you for your suggestions. In this "data note" type article, we give some preliminary inferences about the results. We will take this as a clue for more in-depth research in the future. At the same time, we also hope to provide data and thinking support for the research of other researchers, so as to study the mechanism related to temperature adaptability more thoroughly.<br><br>5.There too many errors in detail.<br>(1)Considering the genome size is smaller than 1Gb, it is not appropriate that 'k=21' was used to estimate the genome size, because it might lead to larger evaluation results. At the same time, the software that calculates kmer needs to be cited.<br>Answer: Thank you for your reminding. According to the formula (4 ^ k / genome > 200) (Kelley et al. 2010), the k-mer distribution diagrams with k = 19 were reconstructed. We have changed the data of genome survey and cited the software that calculated k-mers, please see the Data Description 2 and the Additional File 1.<br>Reference:<br>Kelley DR, Schatz MC, Salzberg SL. Quake: quality-aware detection and correction of sequencing errors. Genome Biology 11, R116 (2010).<br><br>(2)Analysis of gene function annotations is missing.

	Answer: We have added some analysis of gene function annotations. Please see the third paragraph of the conclusion.<br><br>(3)'Finally, the genome sequences with total lengths of 619.41 and 616.63 Mb were attached to the 24 and 21 chromosomes' Please cite relevant literature on karyotype analysis.<br>Answer: We have cited relevant literature on karyotype analysis, please see the Data Description 4.<br><br>(4)'Using diamond v0.9.29.130 [39] to compare the gene sequences of these two species, 21,291 collinear gene pairs were obtained. Using the collinearity of these gene pairs, the collinearity of the linear pattern of C. argus and C. maculata was demonstrated by JCVI v0.9.13 [40] (Fig. 2B).' It is impossible to obtain the result of Fig. 2B by comparing the gene sequences.<br>Answer: Thank you for the comments. We have changed this sentence to “Using blastp to compare the gene protein sequences of these two species, then the genes in all collinearity blocks were obtained, and finally the collinearity map of C. argus and C. maculata was drawn by MCScanX [44] (Fig. 2B).” Please see the Data Description 6.<br><br>(5)If you use the word 'significantly', p-value should be added.<br>Answer: Thank you for your reminding. We have added the P-values.<br><br>(6)'The DEGs at each time point were enriched by GO and KEGG, and the top five items were selected for illustration.' 'top five iterms' doesn't equal 'significantly enriched'. It is meaningless to analyse 'top five iterms' without p-value.<br>Answer: Thank you for reminding us the improper description on the study. We have changed this sentence to “The DEGs at each time point were enriched by GO and KEGG, and the top five items of significantly ($P < 0.05$) enriched items were selected for illustration”. Please see the Data Description 9.<br><br>(7)The number in Additional File 1 and Table 1 needs be formatted '1000 separator(,)'.<br>Answer: Thank you for your reminding. We've formatted it. Please see the number in Additional File 2, 3 and Table 1.<br><br>(8)In Additional File 5, the species name needs to be italicized.<br>Answer: The species names In Additional File 5 were italicized. Did you say additional file 14? We have changed it. Thank you!<br><br>(9)In Additional File 8, the header of table is missing.<br>Answer: Thank you for your reminding. We have added the header of table.<br><br>(10)'Based on this, the box line diagram, PCA map, and cluster diagram of tissue expression were drawn to analyse the overall expression of genes and the correlation between tissues (Additional File 12 and 13).' In Additional File 12&13, 'PCA map' cannot be found.<br>Answer: Sorry, our quotation is not clear. We have modified it. Please see the Data Description 4.<br><br>(11)'The box line diagram showed that the number of genes detected in the brain tissue of C. argus and C. maculata was significantly higher than that in the liver (Fig. 4B).' In Fig. 4B, 'the box line diagram' cannot be found.<br>Answer: Sorry. We have modified it. Please see the Data Description 4.<br><br>(12)In Additional File 14, 'Alignment information' cannot be found.<br>Answer: Thank you for the comments. Since a gene corresponds to multiple comparison information, it is automatically ignored during statistics. We have added a page to the file to supplement this alignment information.
**Additional Information:**	
**Question**	**Response**
Are you submitting this manuscript to a special series or article collection?	No

Powered by Editorial Manager® and ProduXion Manager® from Aries Systems Corporation

**Experimental design and statistics**<br><br>Full details of the experimental design and statistical methods used should be given in the Methods section, as detailed in our Minimum Standards Reporting Checklist. Information essential to interpreting the data presented should be made available in the figure legends.<br><br>Have you included all the information requested in your manuscript?	Yes
**Resources**<br><br>A description of all resources used, including antibodies, cell lines, animals and software tools, with enough information to allow them to be uniquely identified, should be included in the Methods section. Authors are strongly encouraged to cite Research Resource Identifiers (RRIDs) for antibodies, model organisms and tools, where possible.<br><br>Have you included the information requested as detailed in our Minimum Standards Reporting Checklist?	Yes
**Availability of data and materials**<br><br>All datasets and code on which the conclusions of the paper rely must be either included in your submission or deposited in publicly available repositories (where available and ethically appropriate), referencing such data using a unique identifier in the references and in the "Availability of Data and Materials" section of your manuscript.<br><br>Have you have met the above requirement as detailed in our Minimum Standards Reporting Checklist?	Yes

Manuscript Click here to access/download;Manuscript;Reviesd manuscript.docx

Chromosome-level genome assemblies of *C. argus* and *C. maculata* and comparative analysis of their temperature adaptability

Mi Ou[a, †], Rong Huang[b, †], Cheng Yang[b], Bin Gui[b], Qing Luo[a], Jian Zhao[a], Yongming Li[b], Lanjie Liao[b], Zuoyan Zhu[b], Yaping Wang[b, c, *], Kunci Chen[a, *]

[a]Key Laboratory of Tropical and Subtropical Fishery Resources Application and Cultivation, Ministry of Agriculture, Pearl River Fisheries Research Institute, Chinese Academy of Fishery Sciences, Guangzhou, 510380, China

[b]State Key Laboratory of Freshwater Ecology and Biotechnology, Institute of Hydrobiology, Chinese Academy of Sciences, Wuhan, 430072, China

[c]Innovative Academy of Seed Design, Chinese Academy of Sciences, Beijing, 100101, China

[*]Correspondence: wangyp@ihb.ac.cn (Y. W.); chenkunci@aliyun.com (K. C.)

[†] These authors contributed equally to this work.

Abstract

Background: *Channa argus* and *Channa maculata* are the main cultured species of the family Channidae. The relationship between them is close enough that they can mate, however their temperature adaptability is quite different. **Results:** In this study, we sequenced and assembled the whole genomes of *C. argus* and *C. maculata* for the first time and obtained chromosome-level genome assemblies of 630.39 and 618.82 Mb, respectively. Contig N50 was 13.20 and 21.73 Mb, scaffold N50 was 27.66 and 28.37 Mb, with 28,054 and 24,115 coding genes annotated for *C. argus* and *C. maculata*, respectively. *C. argus* and *C. maculata* have 24 and 21chromosomes, respectively. Three pairs of chromosomes in *C. argus* correspond to three chromosomes in *C. maculate, suggesting three chromosomal fusion events in C. maculate*. Comparative analysis of their gene families showed that some immune-related genes were unique or expandable to *C. maculate*, such as genes related to herpes simplex infection. The transcriptome differences related to temperature adaptation revealed that the brain and liver of *C. argus* rapidly produced more DEGs than *C. maculata*. The genes in the FoxO signalling pathway were significantly enriched in *C. argus* during the cooling process ($P < 0.05$), and the expression of three transcription factor genes in this pathway was significantly different between *C. argus* and *C. maculata* ($P < 0.01$). **Conclusions:** *C. maculata* may have higher resistance to certain diseases, while *C. argus* has a faster and stronger response to low-temperature stress, and thus has better adaptability to a low-temperature environment. This study provides a high-quality genome research platform for follow-up studies of Channidae, and provides important clues for the differences in the low-temperature adaptation of fish.

***Keywords*:** *Channa argus*, *Channa maculata*, Genome, Transcriptome, Low-temperature adaptation

Background

C. argus and *C. maculata* belong to the Perciformes, Channidae, and Channa [1], and are the main cultured species of Channidae. *C. argus* is widely distributed in China, India, and Southeast Asia to the Far East of Russia, North Korea, Japan, and other major water systems with high cold resistance. *C. maculata* is distributed in warm water systems in China, the Philippines, Vietnam, Madagascar, the United States, Japan, and other places, with low cold resistance [2]. In 2019, the

output of Channidae in China reached 460,000 tons [3].

The ability of different fish to adapt to environmental temperature is different, which is the result of long-term adaptation and evolution, and the specific expression of genetic information. At present, the physiological response of fish to low temperature has been deeply studied [4]. At the same time, the molecular biological mechanism of fish adaptation to low temperature environment and tolerance to low temperature stress is in the ascendant, causing extensive interest of researchers [5]. The cost reduction of high-throughput sequencing technology and the application of bioinformatics technology allow researchers to use omics methods to study the molecular signalling pathways of fish under low temperature stress, analyse the molecular mechanism of fish responding to low temperature stress at the overall biological level, and explore the functional genes of low-temperature tolerance [6].

The cold tolerance of fish is an important economic characteristic of the breed, and is related to its growth cycle and extension range [2]. In order to understand the reasons for the difference in cold tolerance between *C. argus* and *C. maculata*, whole genome sequencing and assembly of these two species were carried out in this study. The chromosome-level genomic sequences of the two species were obtained for the first time. Based on this, the transcriptome differences related to temperature adaptation between *C. argus* and *C. maculata* were analysed, providing clues for research on the low-temperature adaptation of fish.

Data Description

1. Source of experimental fish and preparation of DNA

A female *C. argus* and a female *C. maculata*, which were provided by the Pearl River Fisheries Research Institute, Chinese Academy of Fishery Sciences, were dissected to obtain muscle tissue and immediately frozen in liquid nitrogen for storage. The cetyltrimethylammonium bromide method was used to extract DNA from the muscle tissue. 1% agarose gel electrophoresis and Qubit 3.0 (Thermo Fisher Scientific Inc., Massachusetts, USA) were used to detect the quality and concentration of the extracted DNA.

Before the dissection of the experimental fish, the fish were anaesthetized with ethyl 3-aminobenzoate methanesulfonate. The experimental protocol of this study was approved by the Animal Ethics Committee of the Institute of Hydrobiology, Chinese Academy of Sciences

(reference number: Y81F101).

2. Illumina sequencing and genome survey

Two 350 bp libraries were constructed using the *C. argus* and *C. maculata* muscle tissue DNA, and paired-end 150 bp (PE 150) sequencing was performed on the Illumina NovaSeq 6000 platform. The experiment was performed according to the standard protocol provided by Illumina. After the raw data was obtained, 62.90 and 63.90 Gb clean data of *C. argus* and *C. maculata* were obtained by routine filtering. Two k-mer distribution maps with k = 19 were constructed based on clean data using jellyfish v2.1.4 (https://github.com/gmarcais/Jellyfish) (Additional File 1). Based on the distribution of k-mers in *C. argus* and *C. maculata*, it was estimated that the content of repeated sequences was approximately 18.73 and 18.23%, and the heterozygosity was approximately 0.12 and 0.06% using genomescope v1.00 [7], respectively. A total of 49,571,777,400 and 48,531,014,793 k-mers of *C. argus* and *C. maculata* were used for genome length estimation, and the calculated genome lengths were about 658.63 and 652.03 Mb (the formula is k-mer number / average k-mer depth), respectively. In addition, according to the sequencing data analysis, the GC contents of *C. argus* and *C. maculata* genomes were approximately 40.36 and 40.37%, respectively. From the above evaluation results, we inferred that the genomes of *C. argus* and *C. maculata* are both simple genomes.

3. Nanopore sequencing and initial assembly

Two Oxford Nanopore long-read libraries were constructed using *C. argus* and *C. maculata* muscle tissue DNA and sequenced on the Nanopore platform. The process was performed using the Ligation Sequencing Kit 1D (SQK-LSK109) protocol. After filtering low-quality reads and removing the adapters, 118.24 and 101.34 Gb clean data was respectively obtained. The total sequencing depth was approximately 187.57 × and 163.76 ×, the N50 reads were 38.83 and 40.48 Kb, and the average read length was 26.59 and 28.11 Kb for *C. argus* and *C. maculata*, respectively. Using the Canu v1.9 [8], the clean data were corrected, then assembled based on the corrected data using WTDBG v1.2.8 [9], then corrected again with the Nanopore and Illumina sequencing data using the Racon [10] and Pilon v1.23 [11], respectively. Finally, the initial assembled genome sequence of *C. argus* and *C. maculata* had a total length of 630.38 and 618.82

Mb, and contig N50 were 21.50 and 23.25 Mb, respectively. Using BWA [12] to align the Illumina sequencing data with the initial assembled genome, the matching rates were 98.17 and 98.34% (Additional File 2). Busco v2.0 [13] was used to evaluate the integrity of 4,584 conserved core genes in the initial assembled genome, accounting for 96.07 and 97.03%, respectively (Additional File 3), indicating that the initial assemblies were effective.

4. Super assembly based on Hi-C technology

After fixing and cross-linking the *C. argus* and *C. maculata* muscle tissues with formaldehyde, two 300-700 bp Hi-C libraries were constructed according to the methods of Rao et al. [14]. After the libraries were qualified, high-throughput sequencing was performed using an Illumina NovaSeq 6000 with PE150. The raw data were filtered to remove low-quality reads and adapters, and 102.43 and 103.13 Gb clean data for *C. argus* and *C. maculata*, respectively, were obtained. After aligning the clean data with the initial genome assembly, using HiC-Pro v2.11.1 [15] to filter the alignment results, 146,400,814 and 151,732,929 valid interaction pairs were obtained. Based on the valid interaction pairs, the initial genome assemblies were further assembled using the LACHESIS [16], including grouping, sorting, and orientation of the initial assembled sequences. Finally, the genome sequences with total lengths of 619.41 and 616.63 Mb were attached to the 24 and 21 chromosomes [17], respectively, accounting for 98.26 and 99.65% (619,407,135/630,381,055 and 616,629,265/618,815,250), respectively, and the numbers of corresponding sequences were 293 and 227, respectively (Table 1).

Chromosome-level genomes were cut into 100 Kb bins of equal length, the number of Hi-C read pairs covering any two bins was used as the signal of the interaction between the two bins, and two heat maps were drawn to evaluate the assembly quality (Fig. 1A and 1B). The image signal distinguished the 24 and 21 chromosome groups, and the intensity of the interaction at the diagonal position on each chromosome was higher than that at the off-diagonal position, indicating that the assembly effect of chromosomes was strong.

5. Annotation of repetitive sequences, coding genes, and non-coding RNA

Using LTR_FINDER [18] and RepeatScout v1.0.5 [19], two repetitive sequence databases of the genomes were constructed based on the principles of structure prediction and *de novo* prediction,

PASTEClassifier [20] was used to classify the databases. These were then merged with the Repbase database [21] as the final repetitive sequence databases. RepeatMasker v4.0.9 [22] was then used to predict the repetitive sequences of the genomes based on the constructed repetitive sequence databases, repetitive sequences of 117.49 and 118.99 Mb were obtained from *C. argus* and *C. maculata*, respectively (Additional File 4).

Genscan [23], Augustus v2.4 [24], GlimmerHMM v3.0.4 [25], GeneID v1.4 [26], and SNAP v2006-07-28 [27] were used for *de novo* prediction of coding genes. GeMoMa v1.3.1 [28, 29] was used for predictions based on homologous species. Hisat v2.0.4 [30] and Stringtie v1.2.3 [31] were used to assemble transcripts with reference sequences, and TransDecoder v2.0 [32] and GeneMarkS-T v5.1 [33] were used to perform gene prediction. PASA v2.0.2 [34] was used to predict unigene sequences based on transcriptome data without reference sequences. EVM v1.1.1 [35] was used to integrate the prediction results obtained from the above methods and was modified with PASA v2.0.2. Finally, 28,054 and 24,115 coding genes in *C. argus* and *C. maculata* were predicted (Additional File 5). The number of genes supported by the three prediction methods *ab initio*, homology, and RNAseq was 20,544 and 19,990, accounting for 73.23% (20,544/28,054) and 82.90% (19,990/24,115) for *C. argus* and *C. maculata*, respectively.

Different strategies have been used to predict different non-coding RNAs according to the structural characteristics of different non-coding RNAs. Using the Rfam database [36], Blastn was used to perform genome-wide alignment to identify miRNAs and rRNAs. tRNA was identified using tRNAscan-SE v2.0 [37]. Finally, a total of 554 and 247 miRNAs, 1,136 and 633 rRNAs, and 4,172 and 1,784 tRNAs were predicted in *C. argus* and *C. maculata*, respectively (Additional File 6).

6. Evolutionary analysis of the whole genomes

From amphibians to mammals, genome data of 14 species (including *C. argus* and *C. maculata*) with different evolutionary relationships were collected. Using Orthofinder v2.3.7 [38], the protein sequences of these 14 species were classified into families, and the PANTHER v15 database [39] was used to annotate the obtained gene families. A total of 30,269 families were obtained, of which 1,023 were single-copy gene families. A total of 858 families were unique to *C. argus*, and 46 families unique to *C. maculata* (Additional File 7). Using the 1,023 single-copy gene families

and IQ-TREE v1.6.11 [40], the evolutionary tree was constructed using the maximum likelihood (ML) method with the number of bootstraps set to 1,000 and the outgroup set to *Petromyzon marinus*. PAML v4.9i [41] was used to calculate the divergence time, and MCMCtreeR v1.1 [42] was used for evolutionary tree display (Fig. 1C). The genetic relationship between *C. argus* and *C. maculata*, belonging to Perciformes, was the closest, and the differentiation time was 6-44 million years ago (MYA).

Based on the phylogenetic tree with divergence time and the results of gene family clustering, the number of ancestral gene family members of each branch was estimated using CAFE v4.2 [43], so as to predicted the expansion and contraction of the gene family relative to its ancestors ($P < 0.05$) (Additional File 8). The results showed that there were 81 expanded gene families, including 606 genes, and 43 contracted gene families, including 95 genes in *C. argus*, 74 expanded gene families, including 721 genes, and 42 contracted gene families, including 8 genes in *C. maculata*. GO and KEGG enrichment analyses were performed using clusterProfile v3.5.1 (Fig. 2A, Additional File 9). The results showed that there were specific immune pathway-related genes in *C. maculata*, such as the genes involved in the intestinal immune network for lgA production and the genes related to the herpes simplex infection pathway. In addition, the members of the herpes simplex infection gene family in *C. maculata* showed significant expansion ($P < 0.05$).

Using blastp to compare the gene protein sequences of these two species, then the genes in all collinearity blocks were obtained, and finally the collinearity map of *C. argus* and *C. maculata* was drawn by MCScanX [44] (Fig. 2B). Chr 2 and 3 of *C. argus* correspond to Chr 2 of *C. maculata*, Chr 4 and 5 of *C. argus* correspond to Chr 3 of *C. maculata*, and Chr 18 and 19 of *C. argus* correspond to Chr 16 of *C. maculata*. Taking the 24 chromosomes of *C. argus* as a reference, the Hi-C data of *C. argus* and *C. maculata* were mapped to it, and the mapping results confirmed the structural difference (Fig. 3).

7. Low temperature stress and transcriptome sequencing

One-hundred and eighty 2-month-old *C. argus* and *C. maculata* specimens, weighing 86 ± 17 and 56 ± 9 g, were respectively placed in two 700 L of barrels, 90 in each barrel, one was for observation and statistical mortality, while the other was used to collect materials. The fish were kept at 31 °C for 2 weeks. Afterwards, the circulating water-cooling device was connected and

temperature began to decrease (Additional File 10). During this process, the status and mortality of *C. argus* and *C. maculata* were recorded daily (Additional File 10), and a cumulative mortality map was drawn (Fig. 4). *C. argus* began to die at 7 °C, and 34 died at 7 to 2 °C, with a mortality rate of 37.78% (34/90); no death occurred in the following 3 days. *C. maculata* began to die at 8 °C, peaked at 7 °C, and all specimen died at 8 to 4 °C, with a mortality rate of 100% (Fig. 4A). Three *C. argus* and *C. maculata* were randomly selected the first time before cooling (31 °C), and brain and liver tissues were collected from each fish. During the cooling period, the samples were collected again after maintaining 16 °C for 24 h, and the samples were then collected at 10 °C, 8 °C, 6 °C, and 4 °C. The sampling time was before 8:00 (before cooling) per day, and the number and tissue of fish were the same as those of the first time.

After the completion of the low-temperature stress, 72 tissue samples (six time points, three *C. argus* and three *C. maculata*, two tissues per fish) were collected for transcriptome sequencing (PE 150). The sequencing platform was an Illumina NovaSeq 6000, and each sample produced no less than 6 Gb of clean data.

8. Statistics of data and expressed genes in transcriptional sequencing

The data obtained from each tissue is shown in Additional File 11. Using hisat2 [45], clean reads of each tissue were aligned with the genomes of *C. argus* and *C. maculata*. After the initial treatment of gene count matrix by rlogTransformation of DEseq2 [46], the gene expression density map of normalised gene showed that the gene expression in brain and liver tissues of *C. argus* and *C. maculata* was negative binomial (Additional File 12A and 13A).

The transcripts per million (TPM) of each gene were calculated, and the genes of TPM > 1 in all samples were counted. Based on this, the box line diagram, cluster diagram, and PCA map of tissue expression were drawn to analyse the overall expression of genes and the correlation between tissues (Additional File 12B, 12C, 13B, 13C and Fig. 4B). The box line diagram showed that the number of genes detected in the brain tissue of *C. argus* and *C. maculata* was obviously higher than that in the liver (Additional File 12B and 13B). PCA and cluster analysis showed that the difference between the brain and liver in *C. argus* and *C. maculata* was the most significant variable (about 75%) in gene expression, and the change of temperature was the second largest variable in PCA, accounting for 4–6% of the total variable.

9. Differential expression analysis of genes

The number of differentially expressed genes (DEGs) with logFC ≥ 1 at each time point was counted with the gene expression level at control temperature (CT, 31 °C) as the control (Fig. 5). With the decrease in temperature, the number of DEGs in the brain and liver of *C. argus* increased rapidly. At 16 °C, the genes in the brain and liver were obviously upregulated and downregulated. At 4 °C, the number of DEGs in the brain began to decrease; however, the brain response of *C. maculata* was mainly from 10 °C, and the liver response was obviously backward. At 8 °C, the number of DEGs in the brain suddenly decreased to the same level as that at 16 °C, which may be related to the phenotypic characteristics of death and massive shock at 8 °C (Additional File 10).

The DEGs at each time point were enriched by GO and KEGG, and the top five items of significantly ($P < 0.05$) enriched items were selected for illustration. It was found that the functions of DEGs were mainly involved in oxidation-reduction processes, metabolic processes, protein phosphorylation, and the pathways mainly involved the FoxO signalling pathway, cell cycle, focal adhesions, etc. (Fig. 6A and 6B). We noticed that the FoxO signalling pathway only appeared in the top five items in *C. argus*. The FoxO signalling pathway is a transcription factor-related signalling pathway (Fig. 6A). We collected all 88 genes enriched in the FoxO signalling pathway in *C. argus*, the iTAK [47] predicted that 10 of these were transcription factors. According to the collinear relationship of genes between *C. argus* and *C. maculata*, we identified 10 corresponding genes in *C. maculatus* (Additional File 14). Transcriptome data were used to analyse the expression changes of 10 transcription factor genes during the cooling process, and it was found that three showed very significant differences between *C. argus* and *C. maculata* ($p < 0.01$) (Fig. 6C). It is speculated that they may be involved in the regulation of cold tolerance traits in *C. argus*.

Conclusion

In this study, we sequenced the whole genome of two Channidae fish, *C. argus* and *C. maculata*, and assembled genome sequences at the chromosome-level, which can provide a high-quality genome research platform for follow-up research. Genome comparison analysis revealed that *C. maculata* uniquely contains genes involved in the intestinal immune network for lgA production

and the herpes simplex infection pathway. In addition, members of the herpes simplex infection gene family also have a significant expansion in *C. maculata*. Compared with *C. argus*, *C. maculata* may have higher resistance to disease, especially herpes simplex infection.

There are three pairs of chromosomes in *C. argus* which correspond to three chromosomes in *C. maculata*. The median number of chromosomes in fish is generally 24 [48-50]. Therefore, we speculate that the chromosomes of *C. maculata* fused compared with that of *C. argus*.

This study carried out transcriptome analysis to analyse why the cold tolerance of *C. argus* is better than that of *C. maculata*. It is found that both *C. argus* and *C. maculata* had obvious up-regulation and down-regulation responses in oxidation-reduction processes, metabolic processes, protein phosphorylation and other pathways, representing the core molecular response to low temperature exposure. However, the difference was that the brain and liver of *C. argus* quickly produced more DEGs, indicating that the response of *C. argus* to low temperature was faster and stronger than that of *C. maculata*. Transcriptional regulation is a direct response to cold environment for organisms. Cold-adapted fish rely on special strategies to acclimate to cold condition, such as protein biosynthesis, energy metabolism, immune system, lipid metabolism, and signaling pathways, whereas these strategies have been proved to be species-specific [51]. A transcription factor-related signaling pathway, the FoxO signaling pathway, was significantly enriched in *C. argus* ($P < 0.05$) (Fig. 6A). The FoxO family of transcription factors regulates the expression of genes in cellular physiological events including apoptosis, cell-cycle control, glucose metabolism, oxidative stress resistance, and longevity [52]. Three genes in this pathway showed significant differential expression between *C. argus* and *C. maculata* (Fig. 6C), and their function in low-temperature adaptation requires further accurate verification and analysis.

Data Availability

Genome, annotation files and raw sequences for genome assembly including Illumina, Nanopore and Hi-C reads of *C. argus* have been deposited in the NCBI under accession number PRJNA731586, and the corresponding data of *C. maculata* are under accession number PRJNA730430. The transcriptome data related to temperature adaptation of *C. argus* and *C. maculata* were under accession number PRJNA732763.

Additional Files

Additional File 1. K-mer distribution of reads of *C. argus* (A) and *C. maculata* (B). K-mers (k = 19) were extracted from the paired-end library with an insert size of 350 bp. The peak 19-mer depths were 76 (A) and 75 (B), respectively.

Additional File 2. Matching rates of the Illumina sequencing data.

Additional File 3. Integrity of 4,584 conserved core genes.

Additional File 4. Annotation of repetitive sequences.

Additional File 5. Annotation of coding genes.

Additional File 6. Annotation of non-coding RNA.

Additional File 7. Single-copy genes and specific genes in *C. argus* and *C. maculata*.

Additional File 8. Gene family statistics for expansion and contraction.

Additional File 9. GO enrichment analysis of genes in expansion/contraction families. (A) and (B) show the results of *C. argus*, (C) and (D) show the results of *C. maculata*. The abscissa represents GO terms, and the ordinate represents the number and percentage of genes. 10 GO terms with the most significant enrichment were selected and displayed.

Additional File 10. Status and mortality of *C. argus* and *C. maculata* during cooling.

Additional File 11. Data statistics of transcriptome sequencing.

Additional File 12. Preliminary analysis of sequencing data of *C. argus*. (A) The gene expression in brain and liver showed a negative binomial distribution. The abscissa represents the log2 value of the amount of gene expression, and the ordinate represents the percentage. (B) The box line diagram showed that the number of genes detected in brain was higher than that in liver. The abscissa represents the tissue and the ordinate represents the number of genes. (C) Cluster diagram of the brain and liver under different temperatures. Different font colours indicate different temperatures.

Additional File 13. Preliminary analysis of sequencing data of *C. maculata*. (A) The gene expression in brain and liver showed a negative binomial distribution. The abscissa represents the log2 value of the amount of gene expression, and the ordinate represents the percentage. (B) The box line diagram showed that the number of genes detected in brain was higher than that in liver. The abscissa represents the tissue and the ordinate represents the number of genes. (C) Cluster diagram of the brain and liver under different temperatures. Different font colours indicated

different temperatures.

Additional File 14. 10 transcription factor genes in the FoxO signalling pathway of *C. argus* and *C. maculata*.

Abbreviations

DEGs: differentially expressed genes; Gb: gigabase pairs; GC: guanine cytosine; GO: Gene Ontogeny; HPD: highest posterior density; Kb: kilobase pairs; KEGG: Kyoto Encyclopedia of Genes and Genomes; Mb: megabase pairs; ML: maximum likelihood; MYA: million years ago; NCBI: national center for biotechnology information; PCA: principal component analysis; PE: paired end; SRA: sequence read archive; TPM: transcripts per million.

Competing Interests

The authors declare that they have no competing interests.

Funding

This work was supported by the National Key Research & Development Program of China (2018YFD0901201) and the State of Key Laboratory of Freshwater Ecology and Biotechnology (2019FBZ05).

Authors' Contributions

K.C. and Y.W. conceived and designed the experiments. M.O., R.H. and B.G. performed the experiments. C.Y., Q.L., J.Z. and L.L. analyzed the genome and transcriptome data. M.O., R.H. and Y.L. drafted the manuscript. R.H., Y.W., and Z.Z. provided advice on manuscript writing. All authors reviewed the manuscript.

References

1. Pearl River Fisheries Research Institute, Chinese Academy of Fishery Sciences, Shanghai Fisheries University, South China Normal University, etc. Freshwater fishes of Guangdong [M]. Guangzhou: Guangdong Science and Technology Press, 1990:511-514.
2. Ou M, Zhao J, Luo Q, et al. Characteristics of hybrids derived from *Channa argus*

♀ × *Channa maculata* ♂. Aquaculture 2018;**492**:349-56.

3. China Fisheries Statistical Yearbook, 2020.
4. Chen L, Devries AL, Cheng CH. Evolution of antifreeze glycoprotein gene from a trypsinogen gene in Antarctic notothenioid fish. Proc Natl Acad Sci U S A 1997;9**4**(8):3811-6.
5. Beers JM, Jayasundara N. Antarctic notothenioid fish: what are the future consequences of ‘losses’ and ‘gains’ acquired during long-term evolution at cold and stable temperatures? J Exp Biol 2015;**218**(12):1834-45.
6. Volkoff H, Rønnestad I. Effects of temperature on feeding and digestive processes in fish. Temperature (Austin) 2020;**7**(4):307-20.
7. Ranallo-Benavidez TR, Jaron KS, Schatz MC. GenomeScope 2.0 and Smudgeplot for reference-free profiling of polyploid genomes. Nat Commun 2020;**11**(1):1432.
8. Koren S, Walenz BP, Berlin K, et al. Canu: scalable and accurate long-read assembly via adaptive k-mer weighting and repeat separation. Genome Res 2017;**27**(5):722-36.
9. Ruan J, Li H. Fast and accurate long-read assembly with wtdbg2. Nat Methods 2020;**17**:155-8.
10. Vaser R, Ivan S, Nagarajan N, et al. Fast and accurate de novo genome assembly from long uncorrected reads. Genome Res 2017;**27**(5):737-46.
11. Walker BJ, Abeel T, Shea T, et al. Pilon: an integrated tool for comprehensive microbial variant detection and genome assembly improvement. PLoS One 2014;**9**(11):e112963.
12. Li H, Durbin R. Fast and accurate short read alignment with Burrows–Wheeler transform. Bioinformatics 2009;**25**(14):1754-60.
13. Simão FA, Waterhouse RM, Ioannidis P, et al. BUSCO: assessing genome assembly and annotation completeness with single-copy orthologs. Bioinformatics 2015;**31**(19):3210-2.
14. Rao SS, Huntley MH, Durand NC, et al. A 3D map of the human genome at kilobase resolution reveals principles of chromatin looping. Cell 2014;**159**(7):1665-80.
15. Servant N, Varoquaux N, Lajoie BR, et al. HiC-Pro: an optimized and flexible pipeline for Hi-C data processing. Genome Biol 2015;**16**(1):1-11.
16. Burton JN, Adey A, Patwardhan RP, et al. Chromosome-scale scaffolding of de novo genome assemblies based on chromatin interactions. Nature Biotechnol 2013;**31**(12):1119-25.
17. Zhang C, Liu Ni, Yang X, et al. Comparison on karyotype of Minxiangli (*Channa maculata* ♀

× *C. argus* ♂) and its parents. Journal of Shanghai Fisheries University 2005;**14**(2):103-7.

18. Xu Z, Wang H. LTR_FINDER: an efficient tool for the prediction of full-length LTR retrotransposons. Nucleic Acids Res 2007;**35**:265-8.
19. Price AL, Jones NC, Pevzner PA. De novo identification of repeat families in large genomes. Bioinformatics 2005;**21**(1):351-8.
20. Hoede C, Arnoux S, Moisset M, et al. PASTEC: an automatic transposable element classification tool. PLoS One 2014;**9**(5):e91929.
21. Jurka J, Kapitonov VV, Pavlicek A, et al. Repbase update, a database of eukaryotic repetitive elements. Cytogenet Genome Res 2005;**110**(1-4):462-7.
22. Tarailo-Graovac M, Chen N. Using RepeatMasker to identify repetitive elements in genomic sequences. Curr Protoc Bioinformatics 2009;**4**(4):10.
23. Burge C, Karlin S. Prediction of complete gene structures in human genomic DNA. J Mol Biol 1997;**268**:78-94.
24. Stanke M, Waack S. Gene prediction with a hidden Markov model and a new intron submodel. Bioinformatics 2003;**19**:215-25.
25. Majoros WH, Pertea M, Salzberg SL. TigrScan and GlimmerHMM: two open source ab initio eukaryotic gene-finders. Bioinformatics 2004;**20**:2878-9.
26. Blanco E, Parra G, Guigó R. Using geneid to identify genes. Current Protocols in Bioinformatics 2007;**4**(4):3.
27. Korf I. Gene finding in novel genomes. BMC Bioinformatics 2004;**5**:59.
28. Keilwagen J, Wenk M, Erickson JL, et al. Using intron position conservation for homology-based gene prediction. Nucleic Acids Res 2016;**44**(9):e89.
29. Keilwagen J, Hartung F, Paulini M, et al. Combining RNA-seq data and homology-based gene prediction for plants, animals and fungi. BMC Bioinformatics 2018;**19**:189.
30. Kim D, Langmead B, Salzberg SL. HISAT: a fast spliced aligner with low memory requirements. Nat Methods 2015;**12**:357-60.
31. Pertea M, Pertea GM, Antonescu CM, et al. StringTie enables improved reconstruction of a transcriptome from RNA-seq reads. Nat Biotechnol 2015;**33**(3):290-5.
32. Haas BJ, Papanicolaou A. TransDecoder (Find Coding Regions Within Transcripts) http://transdecoder.github.io. Accessed 15 Jan 2020.

33. Tang S, Lomsadze A, Borodovsky M. Identification of protein coding regions in RNA transcripts. Nucleic Acids Res 2015;**43**(12):e78.
34. Campbell MA, Haas BJ, Hamilton JP, et al. Comprehensive analysis of alternative splicing in rice and comparative analyses with *Arabidopsis*. BMC Genomics 2006;**7**:327.
35. Haas BJ, Salzberg SL, Zhu W, et al. Automated eukaryotic gene structure annotation using EVidenceModeler and the Program to Assemble Spliced Alignments. Genome Biol 2008;**9**(1):R7.
36. Griffiths-Jones S, Moxon S, Marshall M, et al. Rfam: annotating non-coding RNAs in complete genomes. Nucleic Acids Res 2005;**33**:D121-4.
37. Lowe TM, Eddy SR. tRNAscan-SE: a program for improved detection of transfer RNA genes in genomic sequence. Nucleic Acids Res 1997;**25**(5):955-64.
38. Emms DM, Kelly S. OrthoFinder: phylogenetic orthology inference for comparative genomics. Genome Biol 2019;**20**(1):238.
39. Mi H, Muruganujan A, Ebert D, et al. PANTHER version 14: more genomes, a new PANTHER GO-slim and improvements in enrichment analysis tools. Nucleic Acids Res 2019;**47**(D1):D419-26.
40. Nguyen LT, Schmidt HA, Von Haeseler A, et al. IQ-TREE: a fast and effective stochastic algorithm for estimating maximum-likelihood phylogenies. Mol Biol Evol 2015;**32**(1):268-74.
41. Yang Z. PAML: a program package for phylogenetic analysis by maximum likelihood. Bioinformatics 1997;**13**(5):555-6.
42. Puttick MN. MCMCtreeR: functions to prepare MCMCtree analyses and visualize posterior ages on trees. Bioinformatics 2019;**35**(24):5321-2.
43. Han MV, Thomas GW, Lugo-Martinez J, et al. Estimating gene gain and loss rates in the presence of error in genome assembly and annotation using CAFE 3. Mol Biol Evol 2013;**30**(8):1987-97.
44. Wang Y, Tang H, DeBarry JD, et al. MCScanX: a toolkit for detection and evolutionary analysis of gene synteny and collinearity. Nucleic Acids Res 2012;**40**(7):e49.
45. Kim D, Paggi JM, Park C, et al. Graph-based genome alignment and genotyping with HISAT2 and HISAT-genotype. Nat Biotechnol 2019;**37**(8):907-15.
46. Love MI, Huber W, Anders S. Moderated estimation of fold change and dispersion for

RNA-seq data with DESeq2. Genome Biol 2014;**15**(12):550.

47. Zheng Y, Jiao C, Sun H, et al. iTAK: A program for genome-wide prediction and classification of plant transcription factors, transcriptional regulators, and protein kinases. Mol Plant 2016;**9**(12):1667-70.
48. Wang Y, Lu Y, Zhang Y, et al. The draft genome of the grass carp (*Ctenopharyngodon idellus*) provides genomic insights into its evolution and vegetarian diet adaptation. Nat Genet 2015;**47**: 625-31.
49. Howe K, Clark MD, Torroja CF, et al. The zebrafish reference genome sequence and its relationship to the human genome. Nature 2013;**496**(7446):498-503.
50. Ren L, Li W, Qin Q, et al. The subgenomes show asymmetric expression of alleles in hybrid lineages of *Megalobrama amblycephala* × *Culter alburnus*. Genome Res 2019;**29**(11):1805-15.
51. Nie M, Tan X, Lu Y, et al. Network of microRNA-transcriptional factor-mRNA in cold response of turbot *Scophthalmus maximus*. Fish Physiol Biochem 2019;**45**(2):583-97.
52. Link W. Introduction to FOXO Biology. Methods Mol Biol 2019;**1890**:1-9.

Figure and table captions

Fig. 1 Genome assembly and evolutionary analysis of *C. argus* and *C. maculata*. The genome wide Hi-C heat maps of *C. argus* (A) and *C. maculate* (B). Chr 1-24 and Chr 1-21 refer to chromosome 1-24 and chromosome 1-21. (C) Evolutionary tree including *C. argus* and *C. maculata*. The black number at each branch represents the divergence time supported by 95% of the highest posterior density (HPD). The top of the tree is absolute age, separated by the shadow of each geological period. The number on the branch shows the number of expanded (red) and contracted (blue) gene families for each clade. The two red asterisks indicate *C. argus* and *C. maculata*.

Fig. 2 Comparative analysis of the *C. argus* and the *C. maculata* genomes. (A) KEGG enrichment analysis of the unique, expansion and contraction gene families. The ordinate is KEGG terms, the abscissa is the number of genes in the pathway, and the colour represents the corresponding p value. On the left is the enrichment result of *C. argus*, and on the right is the enrichment result of *C. maculata*, same asterisks indicate same terms. (B) There was a high collinearity between the

two species. Chr 2 and 3 of *C. argus* correspond to Chr 2 of *C. maculata*, Chr 4 and 5 of *C. argus* correspond to Chr 3 of *C. maculata*, Chr 18 and 19 of *C. argus* correspond to Chr 16 of *C. maculata*.

Fig. 3 Verification of chromosome structure difference between *C. argus* and *C. maculata* genomes. (A) Complete collinearity map. (B) Partial collinearity map showing only the chromosomes with structural differences. (C) Taking the chromosomes of *C. argus* as the reference sequence, the Hi-C data of *C. argus* and *C. maculata* were mapped to it, respectively.

Fig. 4 Low temperature experiment and transcriptome sequencing of *C. argus* and *C. maculata*. (A) Cumulative mortality of *C. argus* and *C. maculata* during cooling. Abscissa represents temperature and ordinate represents cumulative mortality. (B) Principal component analysis (PCA) of expression genes in brain and liver at different temperatures, coordinates are the first three principal components PC1, PC2, and PC3 of PCA, and the scale value represents the contribution of the sample to the principal component.

Fig. 5 Number of DEGs in brain and liver of *C. argus* (A) and *C. maculata* (B) during cooling. The abscissa represents temperature and the ordinate represents the number of genes. Red indicates up-regulated genes and blue indicates down-regulated genes.

Fig. 6 GO and KEGG enrichment analysis of DEGs. The items with noticeable differences between *C. argus* and *C. maculata* were selected for display. (A) Enrichment result in *C. argus* (green for brain, red for liver). The area of the circle indicates the number of genes. (B) Enrichment result in *C. maculata*. (C) The expression of three transcription factor genes in *C. argus* and *C. maculata*. The abscissa represents the tissue samples at different temperatures, and the ordinate represents the expression quantity. The asterisk indicated that the expression level in the *C. argus* was significantly different from that in the *C. maculata* at the same temperature ($P < 0.01$).

Table 1 Summary statistics of the reference genome assemblies of *C. argus* and *C. maculata*.

Table Click here to access/download;Table;Table1.docx

Table 1 Summary statistics of the reference genome assemblies of *C. argus* and *C. maculata*

Species	Assembly	Contig number	Contig length (bp)	Scaffold number	Scaffold length (bp)
C. argus	N50	15	13,290,021	11	27,662,632
	N90	60	1,903,525	22	13,584,876
	Max	-	28,029,688	-	50,138,606
	Total	607	630,381,055	521	630,389,655
	Anchored to chromosomes	-	-	293	619,407,135(98.26%)
C. maculata	N50	13	21,727,292	9	28,367,461
	N90	44	2,420,044	19	21,794,094
	Max	-	26,519,478	-	49,937,344
	Total	338	618,815,250	254	618,823,650
	Anchored to chromosomes	-	-	227	608,076,971(98.61%)

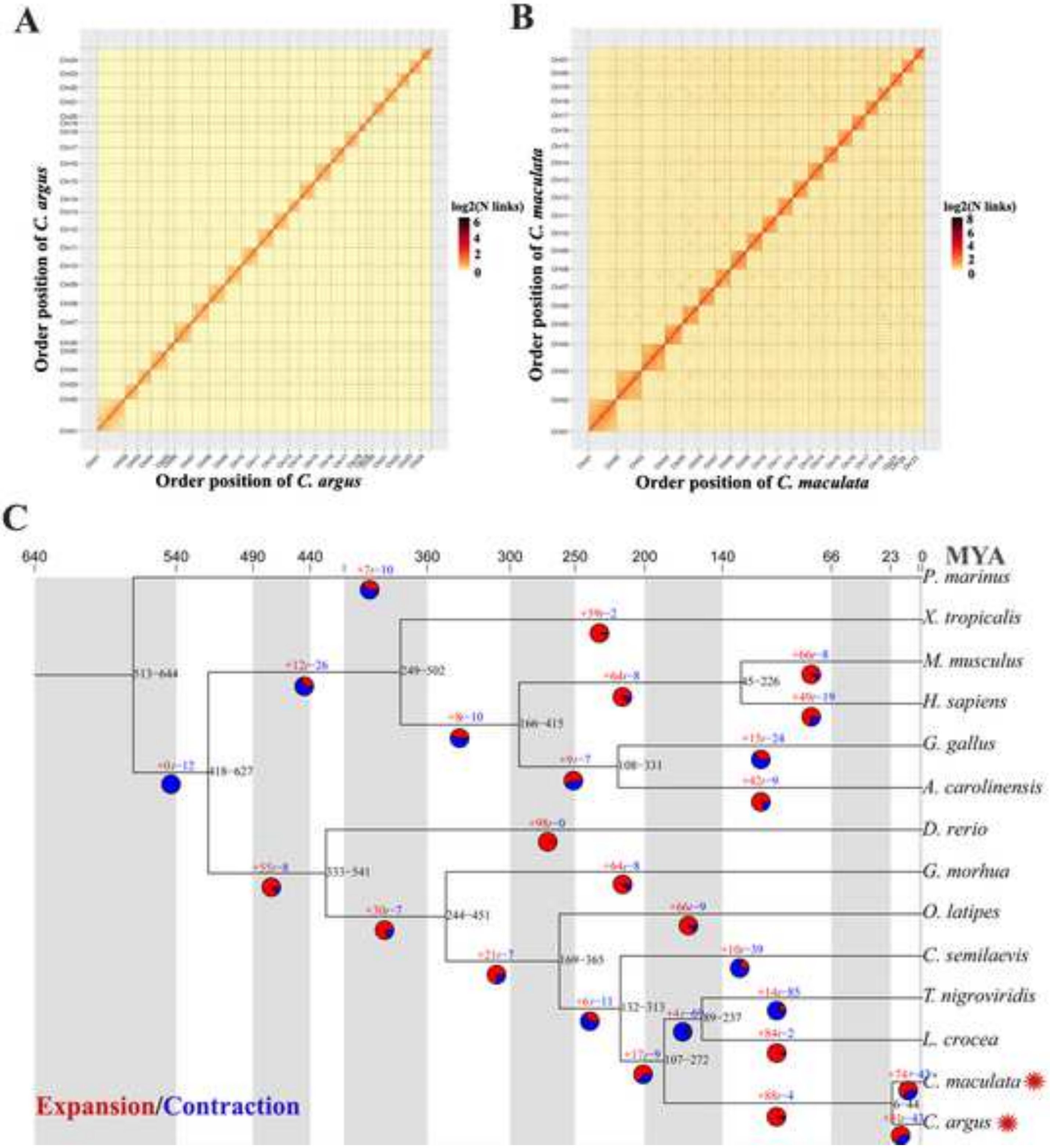
A
Order position of C. argus
Order position of C. argus
log2(N links)
6
4
2
0
B
Order position of C. maculata
Order position of C. maculata
log2(N links)
8
6
4
2
0
C
640 540 490 440 360 300 250 200 140 66 23 0 MYA
P. marinus
X. tropicalis
M. musculus
H. sapiens
G. gallus
A. carolinensis
D. rerio
G. morhua
O. latipes
C. semilaevis
T. nigroviridis
L. crocea
C. maculata
C. argus
Expansion/Contraction

Figure 2

Click here to access/download;Figure;Fig.2.tif

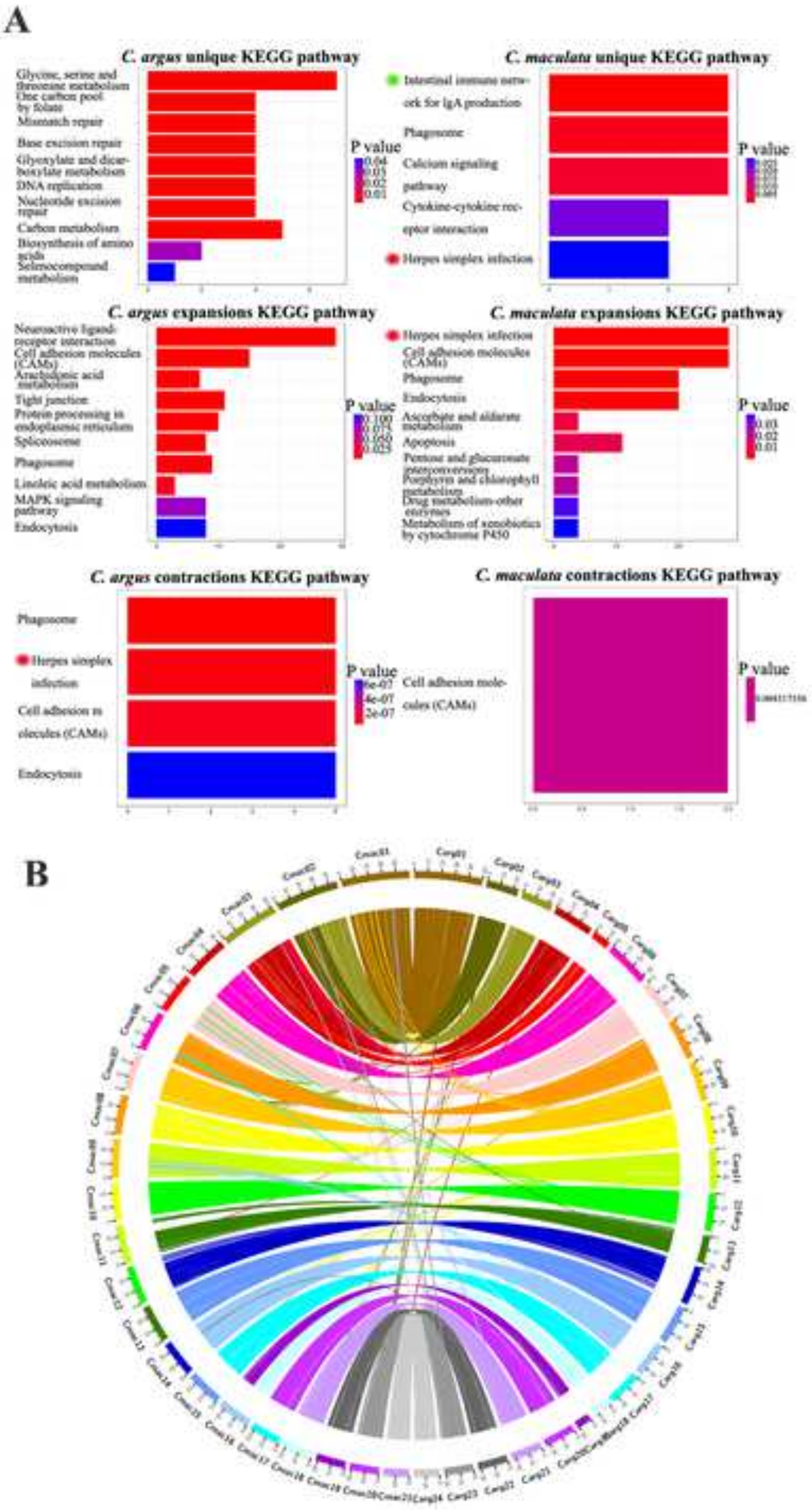

Figure 3 Click here to access/download;Figure;Fig.3.tif

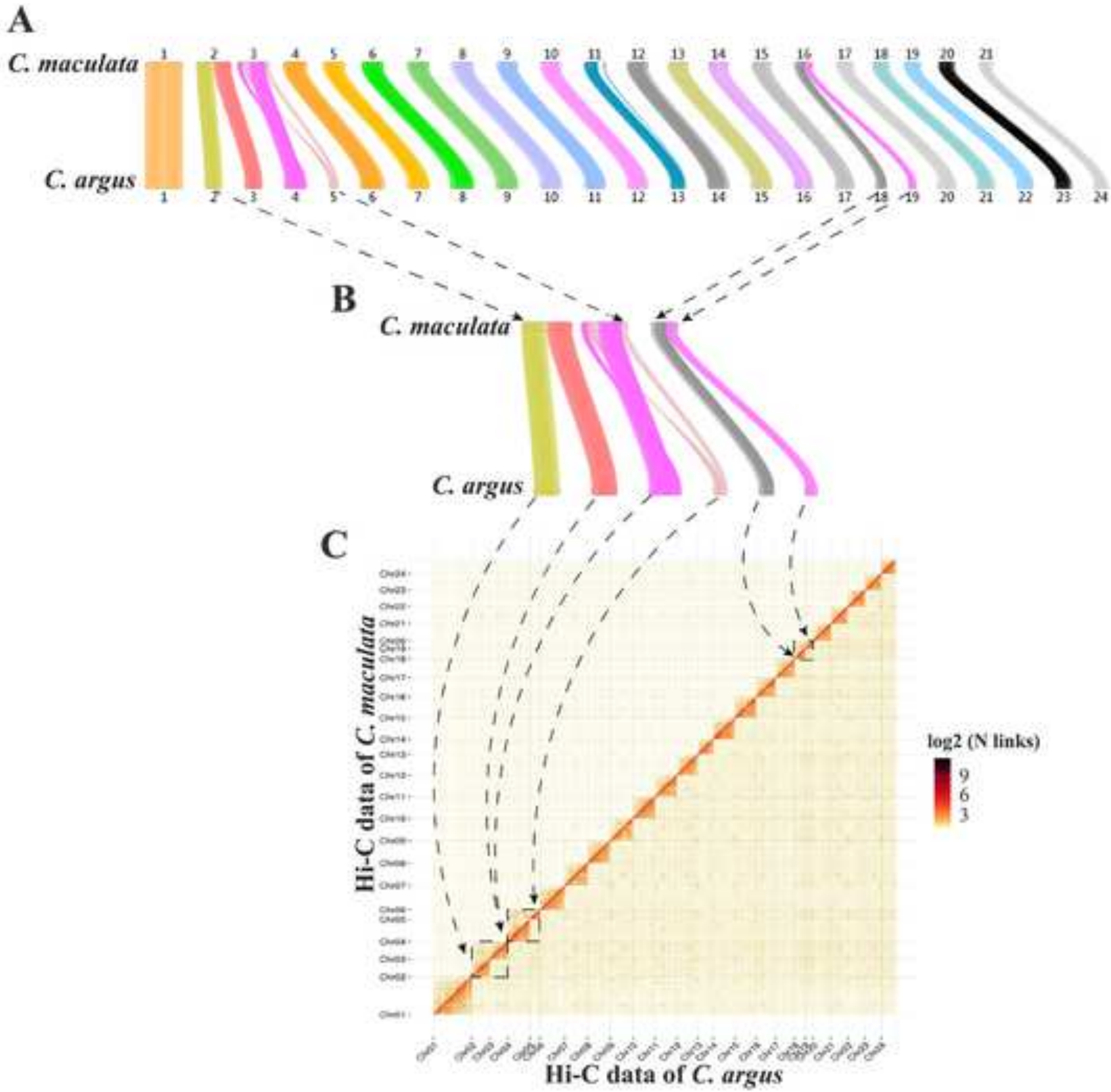

 Click here to access/download;Figure;Fig.4.tif

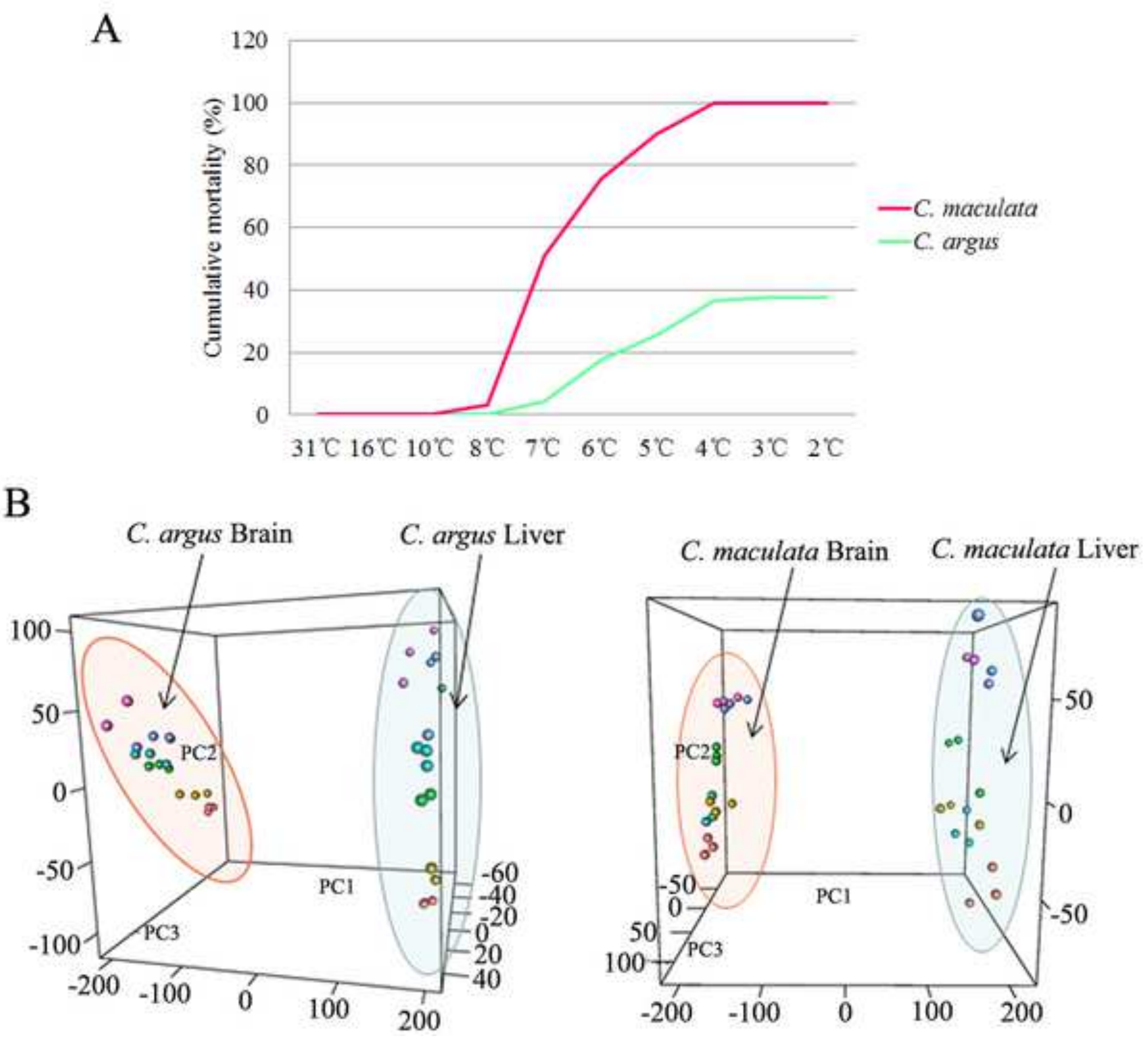

Figure 5

Click here to access/download;Figure;Fig.5.tif

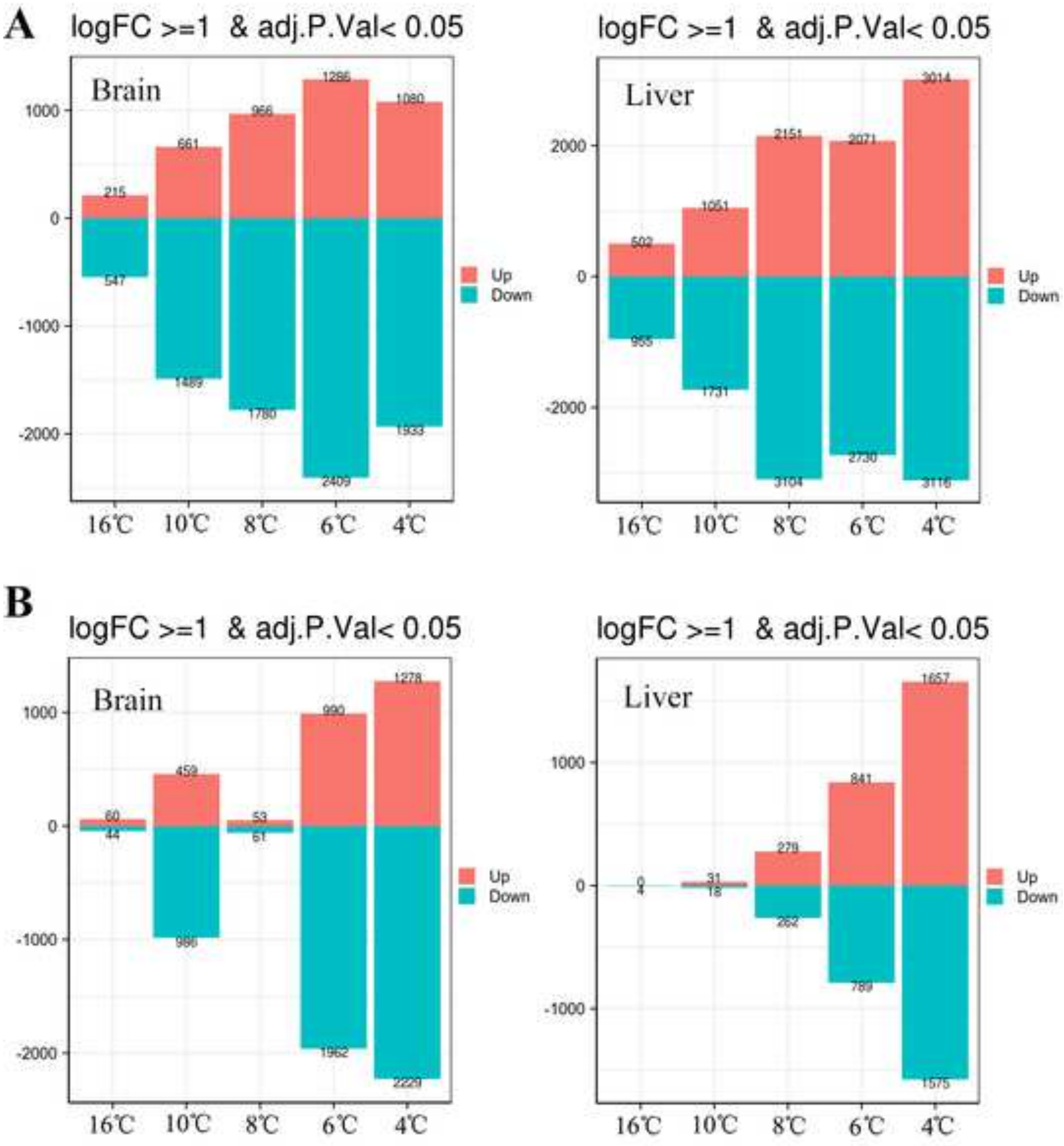

Figure 6

Click here to access/download;Figure;Fig.6.tif

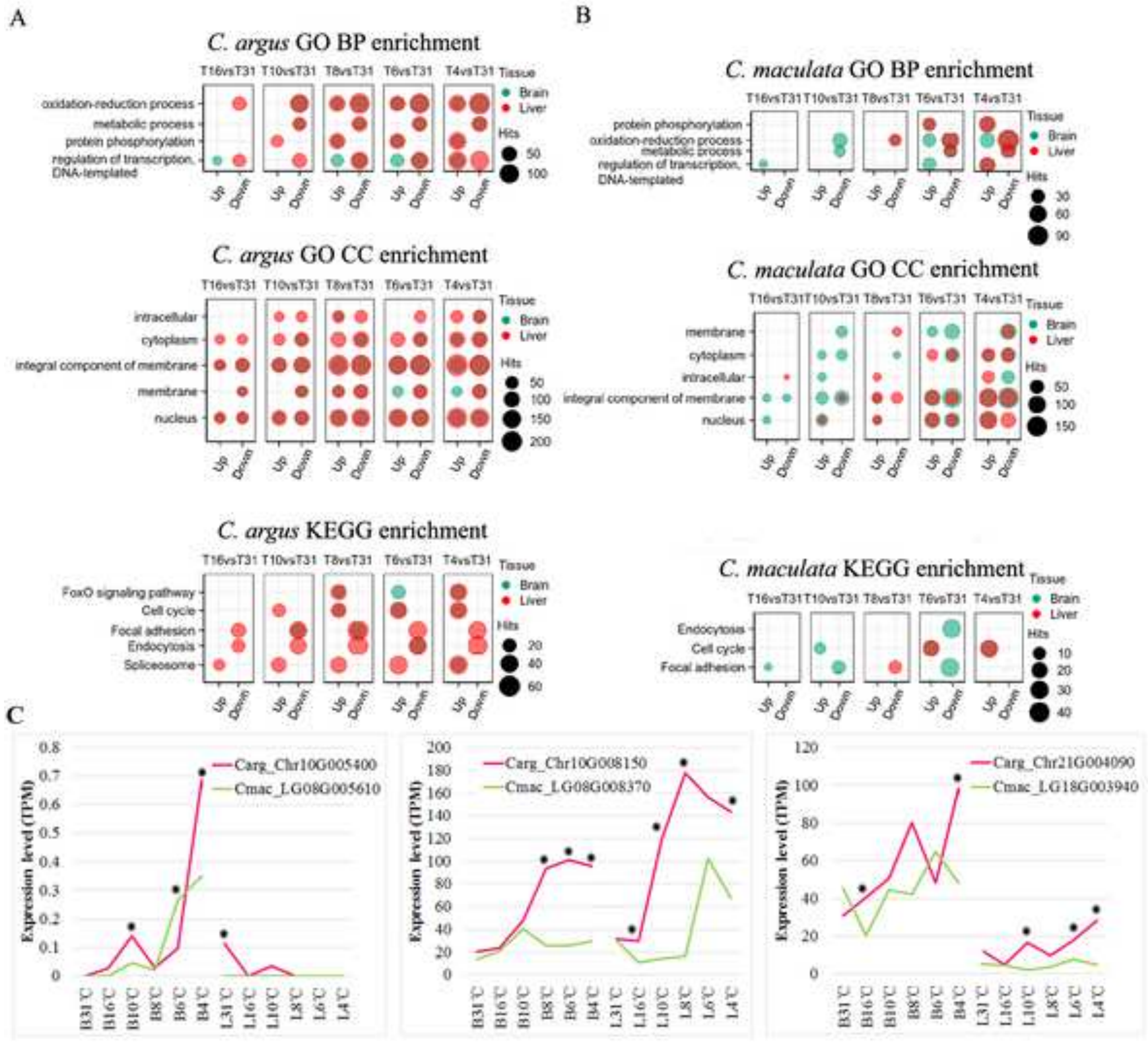

Click here to access/download
Supplementary Material
Additional File 1.tif

Click here to access/download
Supplementary Material
Additional File 2.xlsx

Click here to access/download
Supplementary Material
Additional File 3.xlsx

Click here to access/download
Supplementary Material
Additional File 4.xlsx

Click here to access/download
Supplementary Material
Additional File 5.xlsx

Click here to access/download
Supplementary Material
Additional File 6.xlsx

Click here to access/download
Supplementary Material
Additional File 7.xlsx

Click here to access/download
Supplementary Material
Additional File 8.xlsx

Supplementary Material 9

Click here to access/download
Supplementary Material
Additional File 9.tif

Click here to access/download
Supplementary Material
Additional File 10.xlsx

Click here to access/download
Supplementary Material
Additional File 11.xlsx

Click here to access/download
Supplementary Material
Additional File 12.tif

Supplementary Material 13

Click here to access/download
Supplementary Material
Additional File 13.tif

Click here to access/download

Supplementary Material

Additional File 14.xlsx
