# Supplementary figures and images for: Chromosome-level genome assemblies of Channa argus and Channa maculata and comparative analysis of their temperature adaptability
Source: Gigascience. 2021 Oct 21;10(10):giab070. doi: 10.1093/gigascience/giab070 (PMC8529964; doi:10.1093/gigascience/giab070)

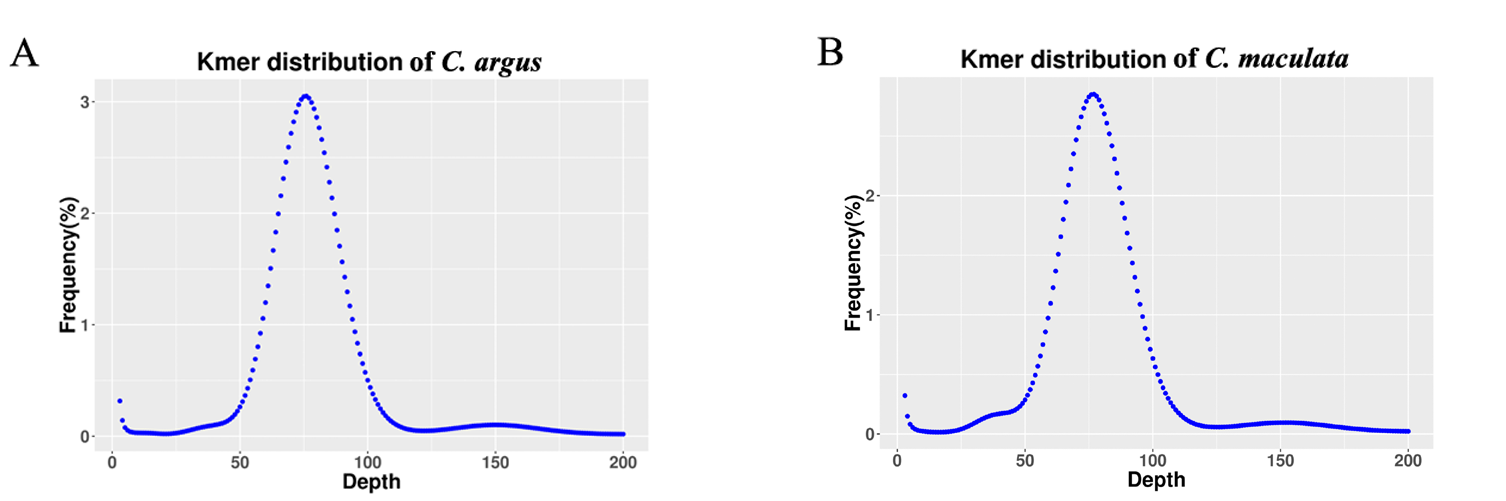

Supplement: giab070_Supplemental_Files [file giab070_supplemental_files.zip › Additional File 1.tif]

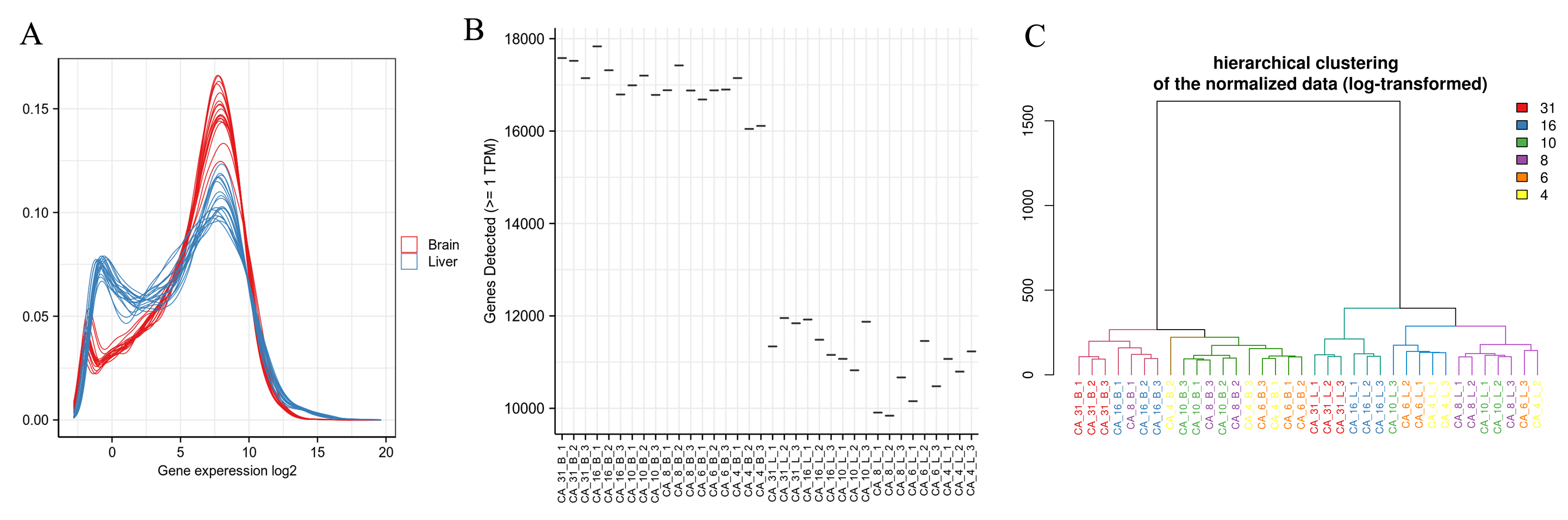

Supplement: giab070_Supplemental_Files [file giab070_supplemental_files.zip › Additional File 12.tif]

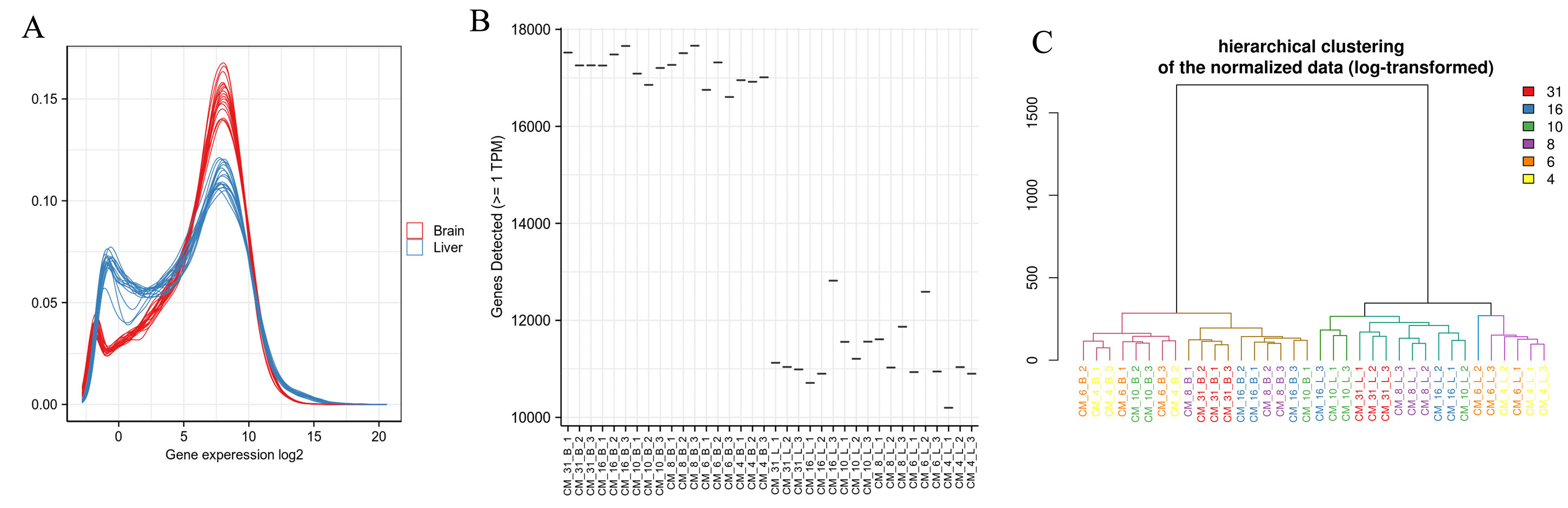

Supplement: giab070_Supplemental_Files [file giab070_supplemental_files.zip › Additional File 13.tif]

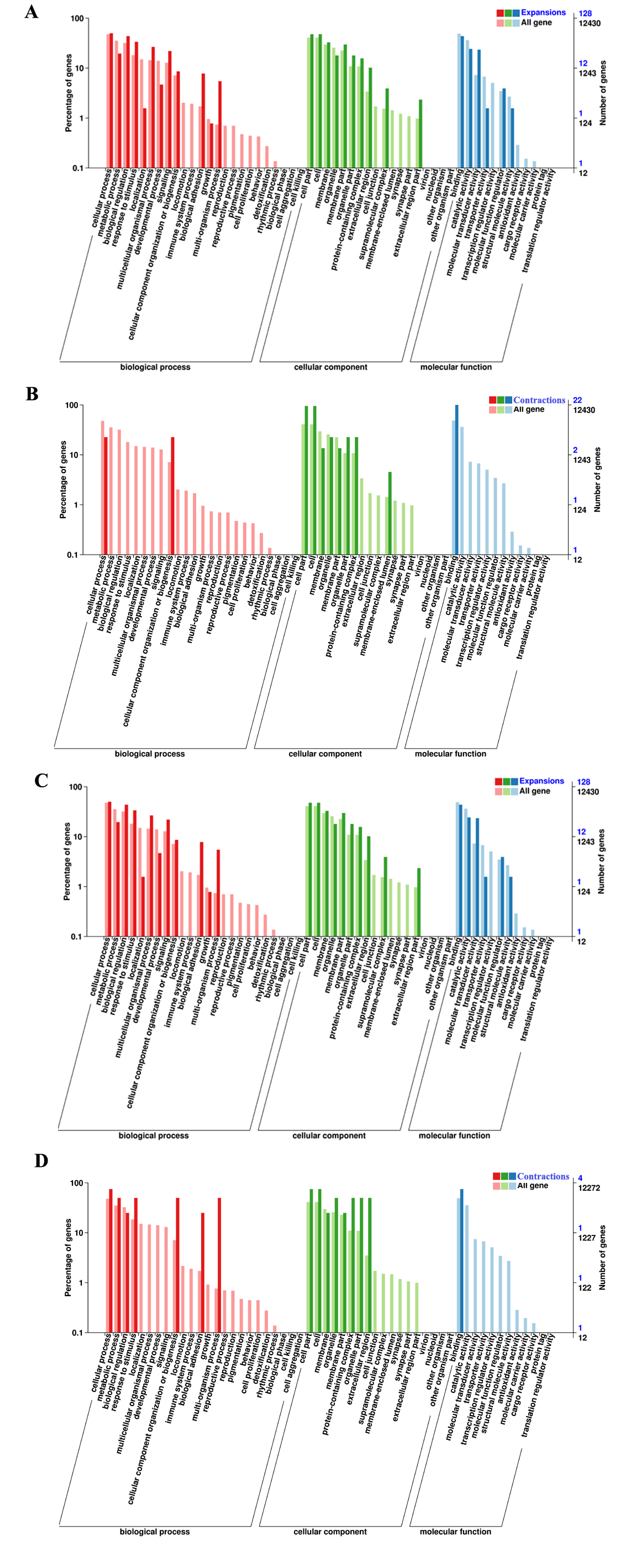

Supplement: giab070_Supplemental_Files [file giab070_supplemental_files.zip › Additional File 9.tif]
