# Supplementary material for: Chromosome-level genome assemblies of Channa argus and Channa maculata and comparative analysis of their temperature adaptability
Source: Gigascience. 2021 Oct 21;10(10):giab070. doi: 10.1093/gigascience/giab070 (PMC8529964; doi:10.1093/gigascience/giab070)
Supplement: giab070_Response_to_Reviewer_Comments_Original_Submission [file giab070_response_to_reviewer_comments_original_submission.pdf]

Author's Response To Reviewer Comments

Close

Reviewer reports:

Reviewer #1: The manuscript: "Chromosome-level genome assemblies of C. argus and C. maculate and comparative analysis of their temperature adaptability" by Ou and Huang et al. provides high quality genomes of two fish species that show differences in their cold tolerance. Using transcriptomic data of tissues collected from fish maintained under cold stress, the authors provide some insights into the pathways that are potentially implicated in this physiological adaptation. I have no major issues with the work and believe it will be of interest to the readers of GigaScience.
Answer: Thank you for your professional review!

Reviewer #2: The manuscript by Ou et al., described the construction of high-quality genomes for two fish species Channa argus and Channa maculata. These species have high economic importance as well as important difference in adaptation to cold. This manuscirpt is valuable because of the high-quality of the genome assembilies, intresting differences in the chromosomal structures (with three independent fusion events), and important phsiological differences of these two species. The publication of this manuscript would facilitate further research on molecular mechanisms underlying the differential adaptation to cold. However, before the paper is accepted for publication, many minor errors, most of which were typos and grammatical errors, need to be addressed.

1. "Genomic collinearity showed that three pairs of chromosomes in C. argus correspond to three chromosomes in C. maculata." This sentence is unclear. "Based on the above results, it is speculated that the chromosomes of C. maculata fused during evolution". This sentence is not supported. Should be modified as something like this: C. maculata and C. argus have 21 and 24 chromosomes, respectivley. Three pairs of chromosomes in C. argus correspond to three chromosomes in C. maculata, suggesting three chromsomal fusion events in C. maculata, or three chromosomal splitting in C. argus.
Answer: Thank you for your suggestion and patient modification! We have revised it according to the suggestion. Please see the blue font section of the abstract.

2. "A female C. argus and a female C. maculata provided by the Pearl River Fisheries Research Institute, Chinese Academy of Fishery Sciences, were dissected to obtain muscle tissue and immediately frozen in liquid nitrogen for storage." Should be modified as: ""A female C. argus and a female C. maculata, which were provided by the Pearl River Fisheries Research Institute, Chinese Academy of Fishery Sciences, were dissected to obtain muscle tissue and immediately frozen in liquid nitrogen for storage."
Answer: We have revised it according to the suggestion. Please see the Data Description 1.

3. "The experimental procedure was performed according to the standard protocol provided by Illumina." Should be modified as: "The experiment was performed according to the standard protocol provided by Illumina."
Answer: We have revised it according to the suggestion. Please see the Data Description 2.

4. Contig -> contig
Answer: Thank you for your reminding. We have revised it according to the suggestion. Please see the Data Description 3.

5. "From amphibians to mammals, 14 species (including C. argus and C. maculata) with different evolutionary degrees were collected". Should be modified as "From amphibians to mammals, genome data of 14 species (including C. argus and C. maculata) with different evolutionary relationships were collected."
Answer: We have revised it according to the suggestion. Please see the Data Description 6.

6. alligned -> aligned
Answer: We have revised it according to the suggestion. Please see the Data Description 8.

7. PacBio -> Nanopore
Answer: Thank you for your suggestions. We have revised it according to the suggestion. Please see the Data Availability.

Reviewer #3: This work sounds good. I have some comments as followed.
1.It should be give much more introductions on the research progress of temperature adaptability.
Answer: Thank you for your careful review and suggestions. We have added relevant contents in the background, please see the second paragraph of the background.

2.'PCA and cluster analysis showed that the difference between the brain and liver in C. argus and C. maculata was the most significant variable (about 75%) in gene expression, and the change of temperature was the second largest variable in PCA, accounting for 4-6% of the total variable.' The differences in gene expression between tissues are obvious, and this study needs more detailed information to explain the differences in different temperature groups.
Answer: We have added detailed information to explain the differences in different temperature groups. Please see the third paragraph of the conclusion.

3.'At 8 °C, the number of DEGs in the brain suddenly decreased to the same level as that at 16 °C, which may be related to the phenotypic characteristics of death and massive shock at 8 °C.' The study didn't elucidate the interesting change.
Answer: Thank you for your reminding. This phenomenon is described in additional file 10. We have referenced "Additional file 10" to this place, please see the Data Description 9.

4.'It was found that the brain and liver of C. argus quickly produced more DEGs, indicating that the response of C. argus to low temperature was faster and stronger than that of C. maculata.' 'Three genes in this pathway showed significant differential expression between C. argus and C. maculata, and their function in low-temperature adaptation requires further accurate verification and analysis.' It is too weak to elucidate the difference of temperature adaptability between C. argus and C. maculate just by the number of DEGs and three genes which are not directly associated with temperature adaptability.
Answer: Thank you for your suggestions. In this "data note" type article, we give some preliminary inferences about the results. We will take this as a clue for more in-depth research in the future. At the same time, we also hope to provide data and thinking support for the research of other researchers, so as to study the mechanism related to temperature adaptability more thoroughly.

5.There too many errors in detail.
(1)Considering the genome size is smaller than 1Gb, it is not appropriate that 'k=21' was used to estimate the genome size, because it might lead to larger evaluation results. At the same time, the software that calculates kmer needs to be cited.
Answer: Thank you for your reminding. According to the formula (4 ^ k / genome > 200) (Kelley et al. 2010), the k-mer distribution diagrams with k = 19 were reconstructed. We have changed the data of genome survey and cited the software that calculated k-mers, please see the Data Description 2 and the Additional File 1.
Reference:
Kelley DR, Schatz MC, Salzberg SL. Quake: quality-aware detection and correction of sequencing errors. Genome Biology 11, R116 (2010).

(2)Analysis of gene function annotations is missing.
Answer: We have added some analysis of gene function annotations. Please see the third paragraph of the conclusion.

(3)'Finally, the genome sequences with total lengths of 619.41 and 616.63 Mb were attached to the 24 and 21 chromosomes' Please cite relevant literature on karyotype analysis.
Answer: We have cited relevant literature on karyotype analysis, please see the Data Description 4.

(4)'Using diamond v0.9.29.130 [39] to compare the gene sequences of these two species, 21,291 collinear gene pairs were obtained. Using the collinearity of these gene pairs, the collinearity of the linear pattern of C. argus and C. maculata was demonstrated by JCVI v0.9.13 [40] (Fig. 2B).' It is impossible to obtain the result of Fig. 2B by comparing the gene sequences.
Answer: Thank you for the comments. We have changed this sentence to “Using blastp to compare the gene protein sequences of these two species, then the genes in all collinearity blocks were obtained, and finally the collinearity map of C. argus and C. maculata was drawn by MCScanX [44] (Fig. 2B).” Please see the Data Description 6.

(5)If you use the word 'significantly', p-value should be added.
Answer: Thank you for your reminding. We have added the P-values.

(6)'The DEGs at each time point were enriched by GO and KEGG, and the top five items were selected for illustration.' 'top five iterms' doesn't equal 'significantly enriched'. It is meaningless to analyse 'top five iterms' without p-value.
Answer: Thank you for reminding us the improper description on the study. We have changed this sentence to "The DEGs at each time point were enriched by GO and KEGG, and the top five items of significantly ($P < 0.05$) enriched items were selected for illustration". Please see the Data Description 9.

(7)The number in Additional File 1 and Table 1 needs be formatted '1000 separator(,)'.
Answer: Thank you for your reminding. We've formatted it. Please see the number in Additional File 2, 3 and Table 1.

(8)In Additional File 5, the species name needs to be italicized.
Answer: The species names In Additional File 5 were italicized. Did you say additional file 14? We have changed it. Thank you!

(9)In Additional File 8, the header of table is missing.
Answer: Thank you for your reminding. We have added the header of table.

(10)'Based on this, the box line diagram, PCA map, and cluster diagram of tissue expression were drawn to analyse the overall expression of genes and the correlation between tissues (Additional File 12 and 13).' In Additional File 12&13, 'PCA map' cannot be found.
Answer: Sorry, our quotation is not clear. We have modified it. Please see the Data Description 4.

(11)'The box line diagram showed that the number of genes detected in the brain tissue of C. argus and C. maculata was significantly higher than that in the liver (Fig. 4B).' In Fig. 4B, 'the box line diagram' cannot be found.
Answer: Sorry. We have modified it. Please see the Data Description 4.

(12)In Additional File 14, 'Alignment information' cannot be found.
Answer: Thank you for the comments. Since a gene corresponds to multiple comparison information, it is automatically ignored during statistics. We have added a page to the file to supplement this alignment information.

Close
