# Supplementary material for: Chromosome-level genome assemblies of Channa argus and Channa maculata and comparative analysis of their temperature adaptability
Source: Gigascience. 2021 Oct 21;10(10):giab070. doi: 10.1093/gigascience/giab070 (PMC8529964; doi:10.1093/gigascience/giab070)
Supplement: giab070_Response_to_Reviewer_Comments_Revision_1 [file giab070_response_to_reviewer_comments_revision_1.pdf]

Author's Response To Reviewer Comments

Close

GIGA-D-21-00172R1
Chromosome-level genome assemblies of C. argus and C. maculata and comparative analysis of their temperature adaptability
Yaping Wang; Mi Ou; Rong Huang; Cheng Yang; Bin Gui; Qing Luo; Jian Zhao; Yongming Li; Lanjie Liao; Zuoyan Zhu; Kunci Chen
GigaScience

Dear Mr. Wang,
Your revised manuscript "Chromosome-level genome assemblies of C. argus and C. maculata and comparative analysis of their temperature adaptability" (GIGA-D-21-00172R1) has been assessed by our reviewer. Based on the review comments, and my own assessment as Editor, I am pleased to inform you that it is potentially acceptable for publication in GigaScience, once you have carried out some essential revisions suggested by our reviewers.

The review report is as below. Please also take a moment to check our website at https://www.editorialmanager.com/giga/ for any additional comments that were saved as attachments.
In addition, please register any new software application in the bio.tools and SciCrunch.org databases to receive RRID (Research Resource Identification Initiative ID) and biotoolsID identifiers, and include these in your manuscript. This will facilitate tracking, reproducibility and re-use of your tool.
Once you have made the necessary corrections, please submit a revised manuscript online at:
https://www.editorialmanager.com/giga/
If you have forgotten your username or password please use the "Send Login Details" link to get your login information. For security reasons, your password will be reset.
Please include a point-by-point within the 'Response to Reviewers' box in the submission system. Please ensure you describe additional experiments that were carried out and include a detailed rebuttal of any criticisms or requested revisions that you disagreed with. Please also ensure that your revised manuscript conforms to the journal style, which can be found in the Instructions for Authors on the journal homepage. If the data and code has been modified in the revision process please be sure to update the public versions of this too.
The due date for submitting the revised version of your article is 02 Nov 2021.
We look forward to receiving your revised manuscript soon.

Best wishes,
Hongfang Zhang
GigaScience
www.gigasciencejournal.com

Answer: We would like to thank you for giving us a chance to resubmit this manuscript, and also thank the reviewers for giving us constructive suggestions which would help us both in English and in depth to improve the quality of the paper. We mark all the changes in red in the revised manuscript.
In addition, all the software used in this manuscript is general software and does not involve the research and development of new software, algorithms and databases. Thank you for your reminder!

Reviewer reports:
Reviewer #3: It is glad to receive the response of comments on this manuscript. Some additions, deletions, and modifications to the content of the manuscript make it more reasonable and exciting. I still have two small comments.

1.Answer: Thank you for the comments. We have changed this sentence to "Using blastp to compare the gene protein sequences of these two species, then the genes in all collinearity blocks were obtained, and finally the collinearity map of C. argus and C. maculata was drawn by MCScanX [44] (Fig. 2B)." Please see the Data Description 6. My previous comment meant that it is usually to compare whole genome synteny two different species including coding region and non-coding region using MUMmer (or LAST,

Lastz, minimap and so on) and Circos (http://circos.ca/) to obtain the result similar to Fig. 2B. According to your method, it is just 'coding genes synteny'.

Answer: Thank you very much for your serious and careful suggestions and tips! The purpose of this collinearity analysis was mainly to find the corresponding relationship among the chromosomes of the two species. Therefore, we selected more conservative coding genes for analysis, and this figure (Fig. 2B) shows the collinearity results of the coding genes. In order to describe more accurately, we have changed this sentence to " Using blastp to compare the gene protein sequences of these two species, then the genes in all collinearity blocks were obtained, and finally the collinearity map of the coding genes of C. argus and C. maculata was drawn by MCScanX [44] (Fig. 2B)". Please see the Data Description 6.

2.Additional File 2. Matching rates of the Illumina sequencing data. 'Mapping rates' may be more commonly used.

Answer: Thank you for your reminder! We have modified it according to your suggestion.

Close
