# Supplementary material for: Chromosome-level genome assemblies of Channa argus and Channa maculata and comparative analysis of their temperature adaptability
Source: Gigascience. 2021 Oct 21;10(10):giab070. doi: 10.1093/gigascience/giab070 (PMC8529964; doi:10.1093/gigascience/giab070)
Supplement: giab070_Response_to_Reviewer_Comments_Revision_2 [file giab070_response_to_reviewer_comments_revision_2.pdf]

Author's Response To Reviewer Comments

Close

GIGA-D-21-00172R2
Chromosome-level genome assemblies of C. argus and C. maculata and comparative analysis of their temperature adaptability
Yaping Wang; Mi Ou; Rong Huang; Cheng Yang; Bin Gui; Qing Luo; Jian Zhao; Yongming Li; Lanjie Liao; Zuoyan Zhu; Kunci Chen
GigaScience

Dear Mr. Wang,

Your manuscript "Chromosome-level genome assemblies of C. argus and C. maculata and comparative analysis of their temperature adaptability" (GIGA-D-21-00172R2) is provisionally accepted pending the final minor changes.

Please submit the revised manuscript with BUSCOv5 and other updated files (if any) to our Editorial Manager system: https://www.editorialmanager.com/giga/. Please also inform us (by email) which files you have updated.

We look forward to receiving your revised manuscript soon.

Best wishes,

Hongfang Zhang
GigaScience
www.gigasciencejournal.com

Answer: Thank you for your processing. We have added the evaluation results of BUSCO v4.0.6, which also corresponds to the result files submitted to GigaDB dataset. In addition, we have updated other changed files. The changed places were marked in red. Please check them.

Close
