# Supplementary material for: Chromosome-level genome assemblies of Channa argus and Channa maculata and comparative analysis of their temperature adaptability
Source: Gigascience. 2021 Oct 21;10(10):giab070. doi: 10.1093/gigascience/giab070 (PMC8529964; doi:10.1093/gigascience/giab070)
Supplement: giab070_Reviewer_2_Report_Original_Submission — Nansheng (Jack) Chen -- 7/7/2021 Reviewed [file giab070_reviewer_2_report_original_submission.pdf]

Reviewer Report

Title: Chromosome-level genome assemblies of C. argus and C. maculata and comparative analysis of their temperature adaptability

Version: Original Submission **Date:** 7/7/2021

Reviewer name: Nansheng (Jack) Chen

Reviewer Comments to Author:

The manuscript by Ou et al., described the construction of high-quality genomes for two fish species Channa argus and Channa maculata. These species have high economic importance as well as important difference in adaptation to cold. This manuscirpt is valuable because of the high-quality of the genome assembilies, intresting differences in the chromosomal structures (with three independent fusion events), and important phsiological differences of these two species. The publication of this manuscript would facilitate further research on molecular mechanisms underlying the differential adaptation to cold. However, before the paper is accepted for publication, many minor errors, most of which were typos and grammatical errors, need to be addressed.

1. "Genomic collinearity showed that three pairs of chromosomes in C. argus correspond to three chromosomes in C. maculata." This sentence is unclear. "Based on the above results, it is speculated that the chromosomes of C. maculata fused during evolution". This sentence is not supported. Should be modified as something like this: C. maculata and C. argus have 21 and 24 chromosomes, respectivley. Three pairs of chromosomes in C. argus correspond to three chromosomes in C. maculata, suggesting three chromsomal fusion events in C. maculata, or three chromosomal splitting in C. argus.
2. "A female C. argus and a female C. maculata provided by the Pearl River Fisheries Research Institute, Chinese Academy of Fishery Sciences, were dissected to obtain muscle tissue and immediately frozen in liquid nitrogen for storage." Should be modified as: ""A female C. argus and a female C. maculata, which were provided by the Pearl River Fisheries Research Institute, Chinese Academy of Fishery Sciences, were dissected to obtain muscle tissue and immediately frozen in liquid nitrogen for storage."
3. "The experimental procedure was performed according to the standard protocol provided by Illumina." Should be modified as: "The experiment was performed according to the standard protocol provided by Illumina."
4. Contig -> contig
5. "From amphibians to mammals, 14 species (including C. argus and C. maculata) with different evolutionary degrees were collected". Should be modified as "From amphibians to mammals, genome data of 14 species (including C. argus and C. maculata) with different evolutionary relationships were collected."
6. alligned -> aligned
7. PacBio -> Nanopore

Level of Interest

Please indicate how interesting you found the manuscript: Choose an item.

Quality of Written English

Please indicate the quality of language in the manuscript: Choose an item.

Declaration of Competing Interests

Please complete a declaration of competing interests, considering the following questions:

- Have you in the past five years received reimbursements, fees, funding, or salary from an organisation that may in any way gain or lose financially from the publication of this manuscript, either now or in the future?
- Do you hold any stocks or shares in an organisation that may in any way gain or lose financially from the publication of this manuscript, either now or in the future?
- Do you hold or are you currently applying for any patents relating to the content of the manuscript?
- Have you received reimbursements, fees, funding, or salary from an organization that holds or has applied for patents relating to the content of the manuscript?
- Do you have any other financial competing interests?
- Do you have any non-financial competing interests in relation to this paper?

If you can answer no to all of the above, write 'I declare that I have no competing interests' below. If your reply is yes to any, please give details below.

I declare that I have no competing interests

I agree to the open peer review policy of the journal. I understand that my name will be included on my report to the authors and, if the manuscript is accepted for publication, my named report including any attachments I upload will be posted on the website along with the authors' responses. I agree for my report to be made available under an Open Access Creative Commons CC-BY license (http://creativecommons.org/licenses/by/4.0/). I understand that any comments which I do not wish to be included in my named report can be included as confidential comments to the editors, which will not be published.

Choose an item.

To further support our reviewers, we have joined with Publons, where you can gain additional credit to further highlight your hard work (see: https://publons.com/journal/530/gigascience). On publication of this paper, your review will be automatically added to Publons, you can then choose whether or not to claim your Publons credit. I understand this statement.

Yes Choose an item.
